# Supplementary material for: IL-1β-mediated adaptive reprogramming of endogenous human cardiac fibroblasts to cells with immune features during fibrotic remodeling
Source: Commun Biol. 2023 Nov 25;6:1200. doi: 10.1038/s42003-023-05463-0 (PMC10673909; doi:10.1038/s42003-023-05463-0)
Supplement: Supplementary file 2 — Supplementary information [file 42003_2023_5463_MOESM2_ESM.pdf]

Supplementary Figures

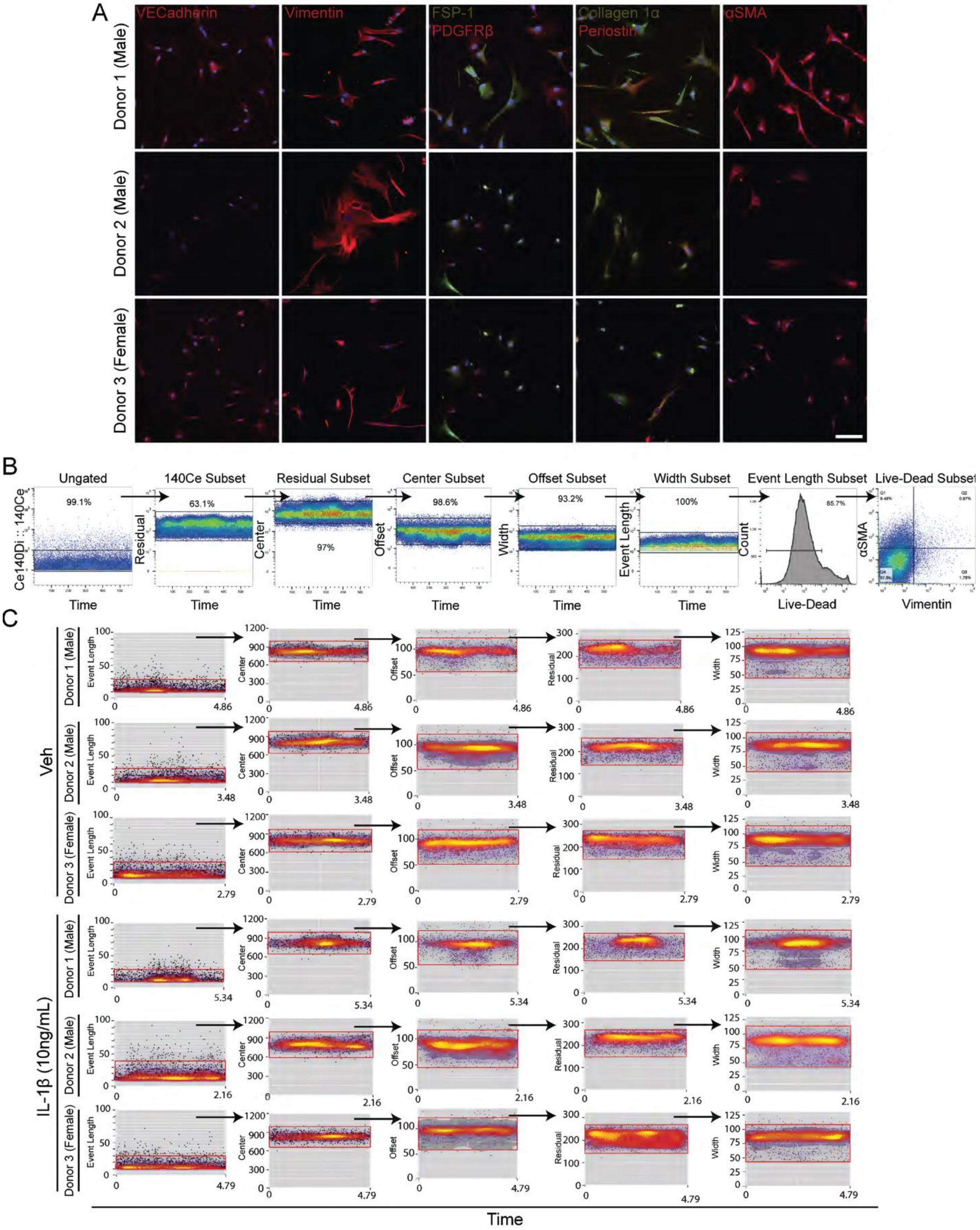

Figure S1: Characterization of primary human ventricular fibroblast and gating strategy for selecting populations for mass cytometry analysis. Immunostaining of passage 3 untreated 20,000 hVCF ((ID# 62122; male, ID# 1281202; male and ID#534282; female) with cardiac fibroblast specific markers; Collagen I (green), Periostin (red), Platelet Derived Growth Factor (PDGFRβ) (red), αSMA (red), Vimentin (red) and Fibroblast Specific Protein (FSP-1) (green). Endothelial cell specific marker,

Vascular Endothelial (VE) Cadherin (red) was used as negative control, Scale bar; 10 µm. (B) Gating strategy for the selection of single nucleated, viable cells used to generate viSNE graphs in Figure 1. Gating is performed on clusters of events with cell staining for two isotopes of Iridium DNA intercalator and viability stain to remove debris and select intact cells. Further gating on the event length parameter was performed to remove cell aggregates to obtain "intact singlets' '. Next, "live cell" gating was employed to select cells with low staining with "thiol" reactive dye. The "event length" parameter refers to the time of detection of a cell event and partially eliminates cell aggregates. From the ungated populations, 140Ce population was gated followed by gating on the residual subset, center subset, offset subset, width subset, event length subset, live-dead subset, and gating of Vim^{+} αSMA^{+} populations. (C) The gating strategy for UMAP analysis using manual and cytofclean tool in R is presented for all the three human donors (ID# 62122; male, ID# 1281202; male and ID#534282; female) untreated or treated with IL-1β (10ng/mL).

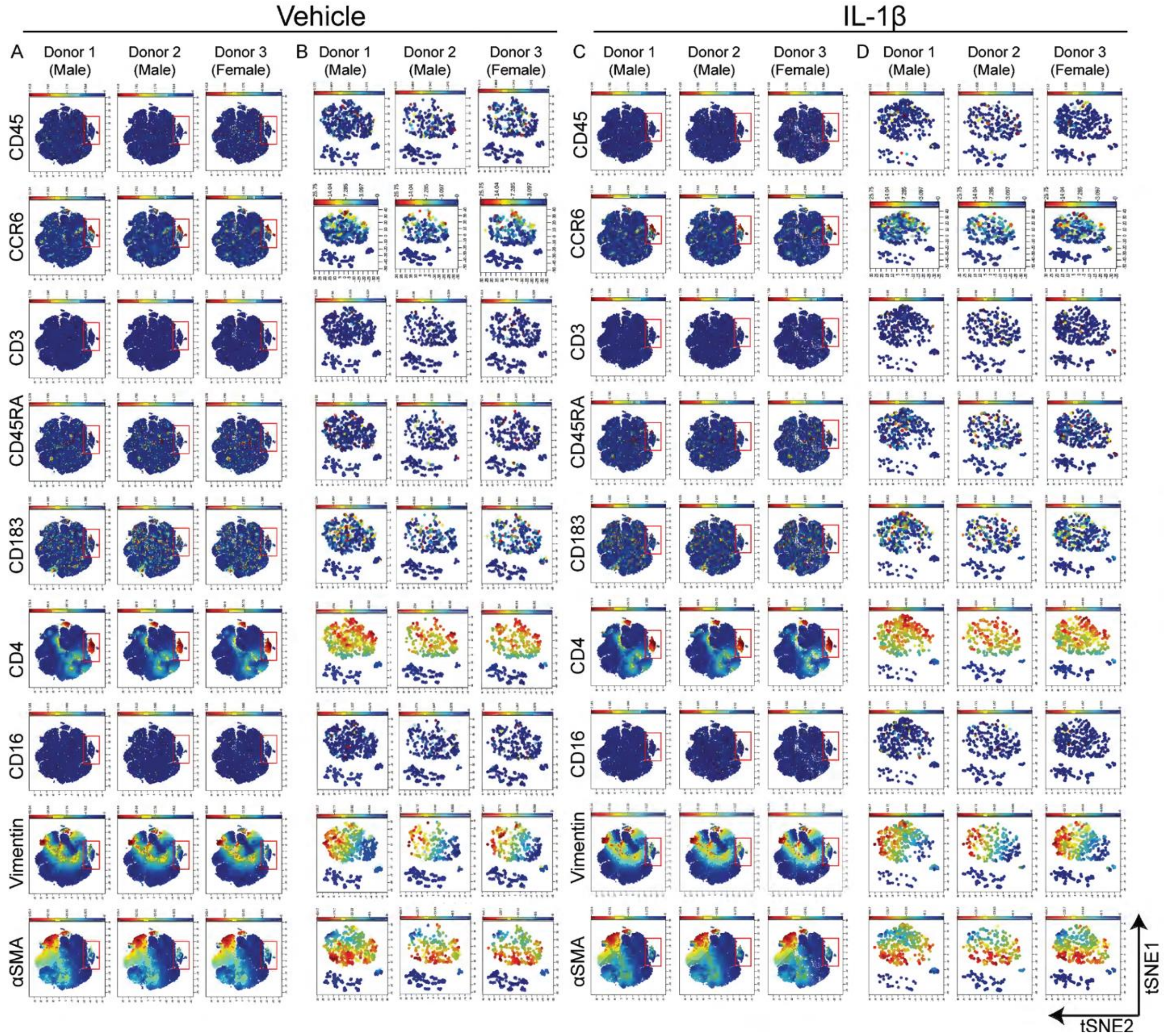

Figure S2: Expression of lymphoid markers on hVCF cell surface in response to interleukin-1-beta. T-distributed Stochastic Neighbor Embedding (tSNE) plots representing the distribution and intensity of markers on primary human ventricular cardiac fibroblasts treated with Veh or IL-1β (10ng/mL) for 96 h and subjected to mass cytometry. (A) tSNE plots of the entire vehicle-treated population of cardiac fibroblast cells expressing CD16, CD4, CD183, CD45RA, CD3, CCR6, and CD45. (B) tSNE plots of the expanded population of cells from A marked by a red square. (C) tSNE plots of the entire IL-1β (10ng/mL) treated population of cardiac fibroblast cells expressing CD16, CD4, CD183, CD45RA, CD3, CCR6, and

CD45. (D) tSNE plots of the expanded population of cells from C marked by a red square. The panels of each donor represent a total 2 x 10^6 cardiac fibroblast cells profiled (n=3, 2 males and 1 female).

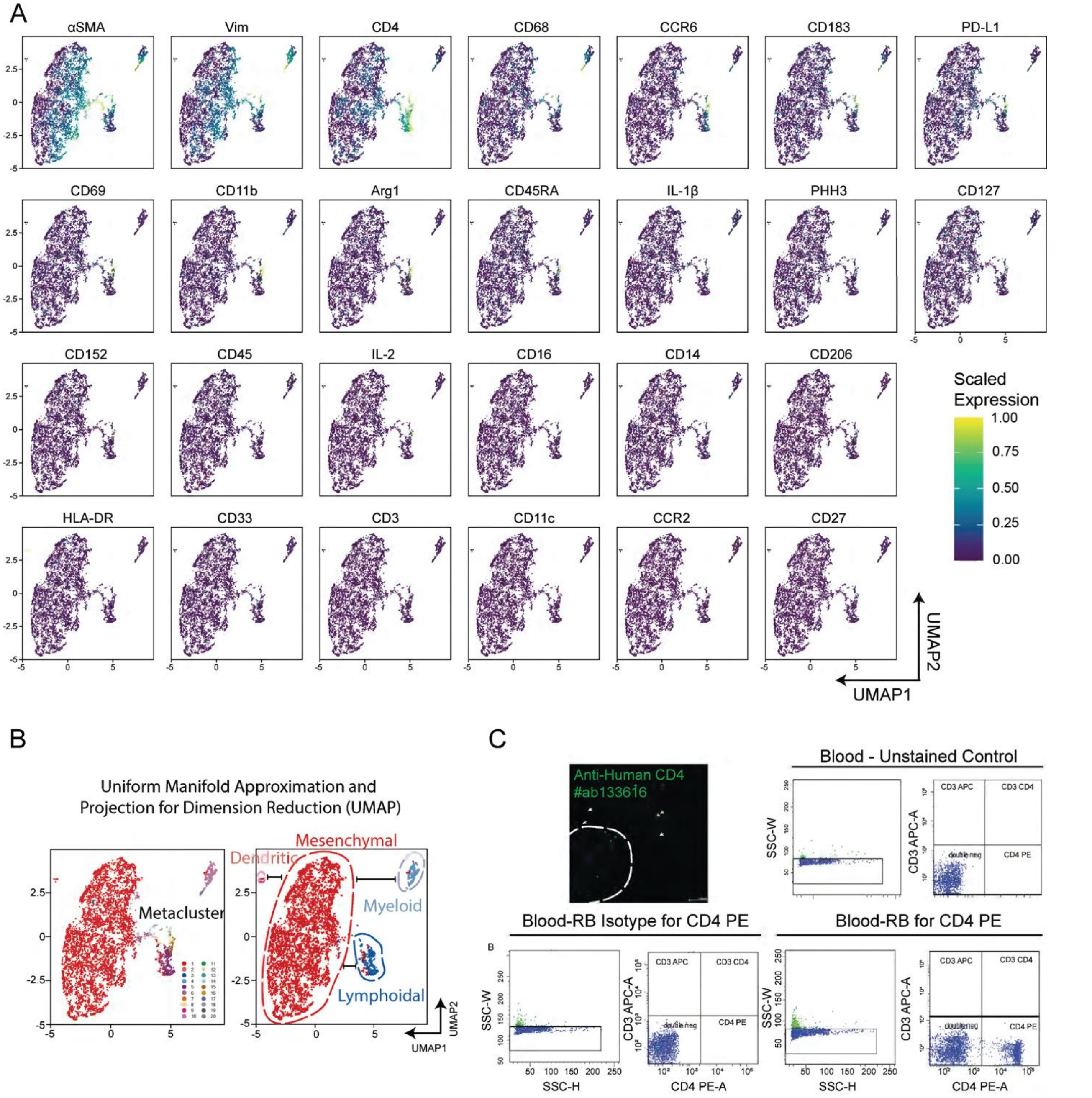

Figure S3: **Uniform manifold approximation and projection algorithm, a non-linear unsupervised learning model used to visualize the mass of all the markers by preserving the global structure**. (A) UMAP, a non-linear unsupervised learning model used to visualize the mass of all the markers by preserving the global structure. The X and Y axis in the UMAP are dimensionless. UMAP allows for the characterization of complex relationships between markers by calculating the distances, neighbors and densities from mass cytometry data using PYTHON and R software tools. The colors stratify similar and dissimilar cell populations through the degree of homogeneity. (B) Meta-clustering of the populations based on the distances of neighbors (UMAP algorithm) to provide a global picture of cell populations. The dotted lines represent the 4 major populations (Lymphoid-, Myeloid-, Mesenchymal- and HLA-positive cells) that were identified in the primary human cardiac fibroblast population. (C) Representative validation image of CD4 antibody from humans and rats used in the experiments reported in this manuscript. The white lines indicate the splenic follicular structure surrounded by the lymphocytes. The

white arrows point to the T cells. Validation of the antibodies using flow cytometry 2D images of rat blood stained with CD4 (ABCAM #ab133616) antibody used against human cells or rat tissue or mice tissue using a negative control and isotype control. Scale bar is 10µm.

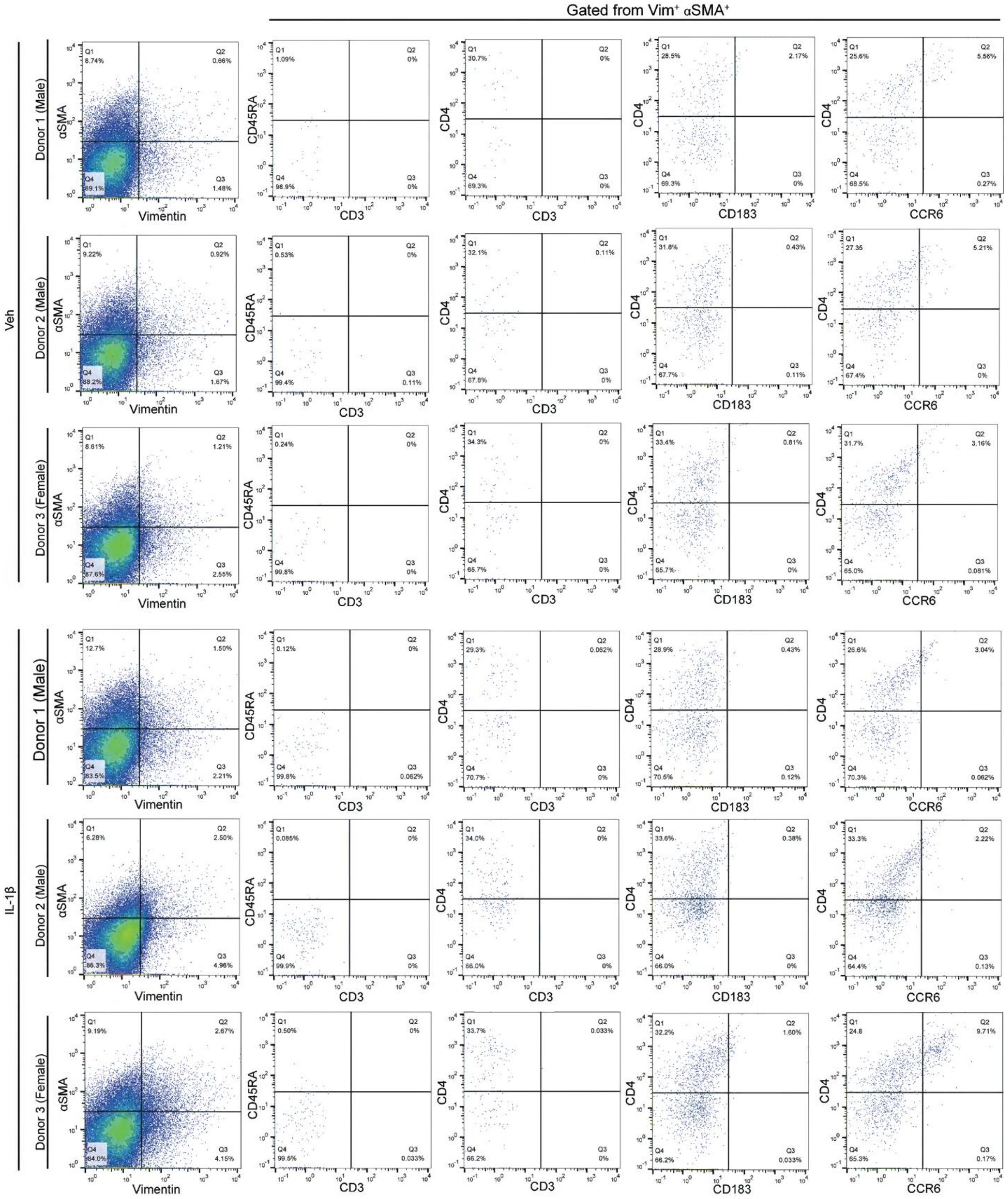

Figure S4: Expression of lymphoid cells markers on cardiac fibroblast cells gated from Vimentin αSMA$^+$ cells. Flow cytometry panels showing the percentage of hVCF expressing lymphoid markers CD3$^+$ CD45RA$^+$, CD3$^+$ CD4$^+$, CD183$^+$ CD4$^+$, CCR6$^+$ CD4$^+$ cells in response to Veh or 48 h IL-1B (10ng/mL) treatment from all the three donors (ID# 62122; male, ID# 1281202; male and ID#534282; female) gated from Vimentin αSMA$^+$ cells. The percentage of cells are indicated in quadrants.

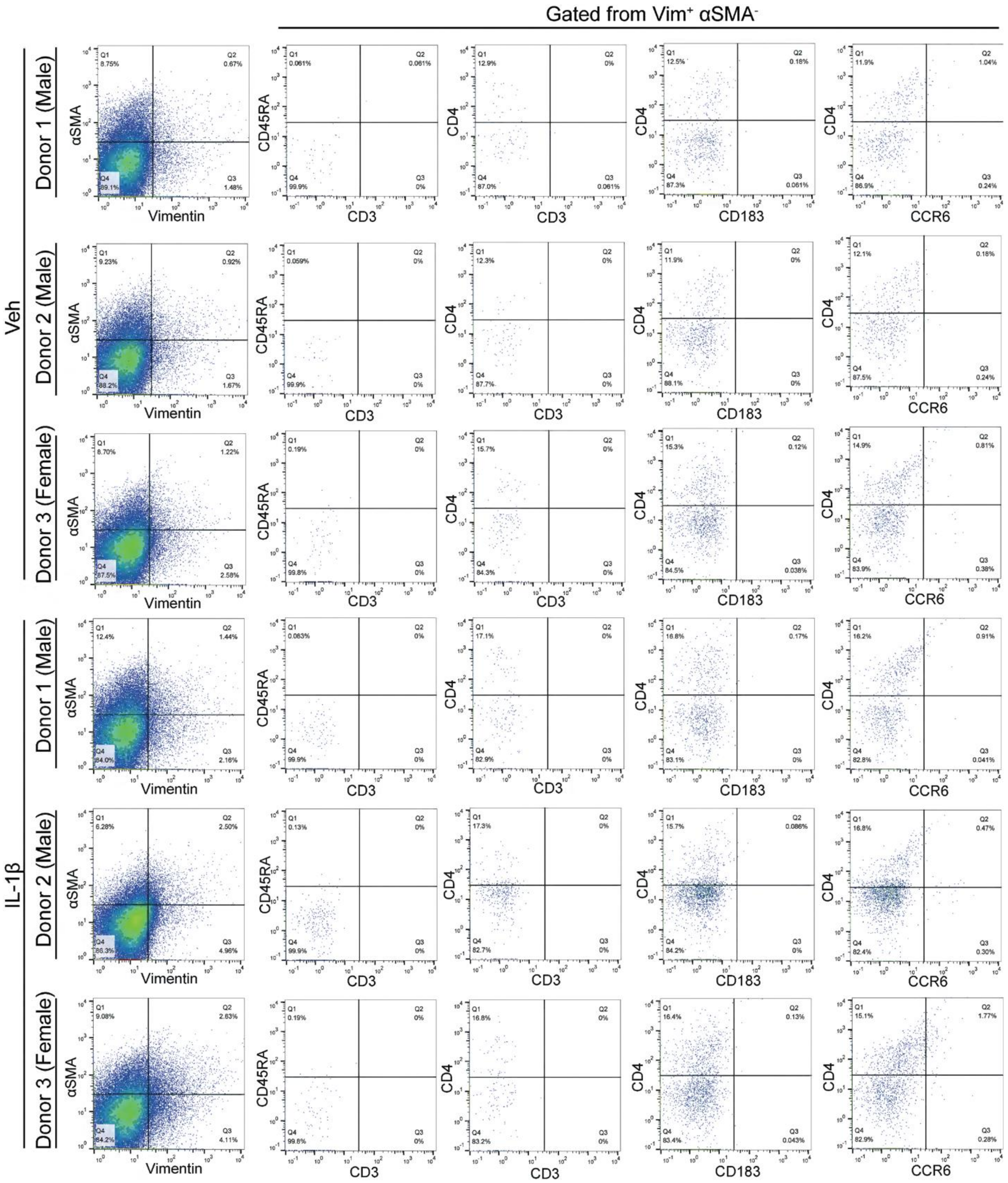

Figure S5: Expression of lymphoid cells markers on cardiac fibroblast cells gated from Vimentin αSMA$^-$ cells. Flow cytometry panels showing the percentage of hVCF expressing lymphoid markers CD3$^+$ CD45RA$^+$, CD3$^+$ CD4$^+$, CD183$^+$ CD4$^+$, CCR6$^+$ CD4$^+$ cells in response to Veh or 48 h IL-1β (10ng/mL) treatment from all the three donors (ID# 62122; male, ID# 1281202; male and ID#534282; female) gated from Vimentin$^+$ αSMA$^-$ cells. The percentage of cells is indicated in quadrants.

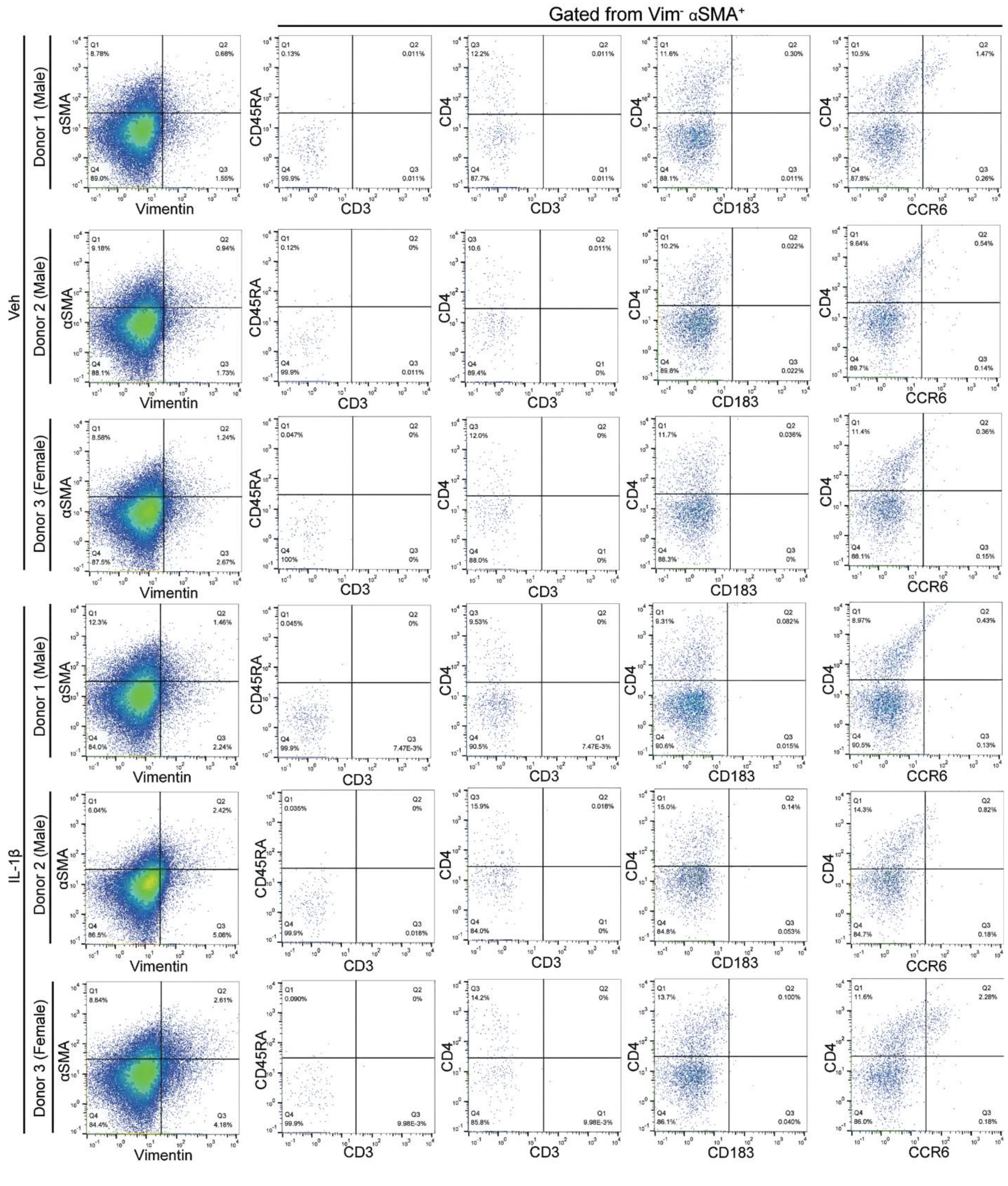

Figure S6: Expression of lymphoid cells markers on cardiac fibroblast cells gated from Vimentin$^-$ αSMA$^+$ cells. Flow cytometry panels showing the percentage of hVCF expressing lymphoid markers CD3$^+$ CD45RA$^+$, CD3$^+$ CD4$^+$, CD183$^+$ CD4$^+$, CCR6$^+$ CD4$^+$ cells in response to Veh or 48 h IL-1β (10ng/mL) treatment from all the three donors (ID# 62122; male, ID# 1281202; male and ID#534282; female) gated from Vimentin$^-$ αSMA$^+$ cells. The percentage of cells are indicated in quadrants.

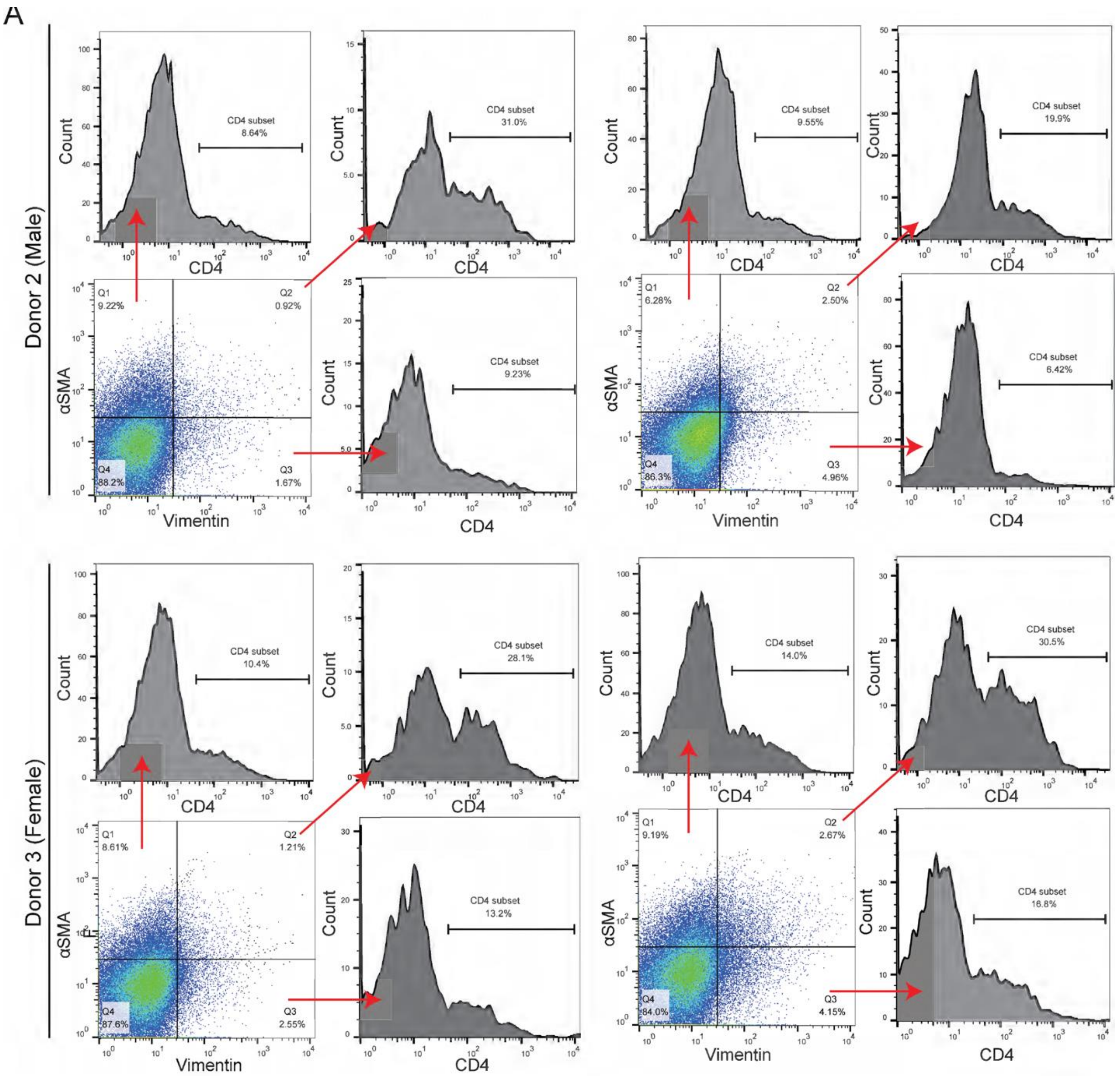

Figure S7: Percentages of CD4 hVCF expressing either Vimentin$^+$ or αSMA$^+$ or both. This supplementary figure is a part of Figure 2 showing histograms of populations of cells expressing Vimentin$^+$ or αSMA$^+$ in response to Veh and IL-1β. Mass cytometry 2D plots of total frequencies of αSMA and Vimentin expressing hVCF. The red arrow from quadrant 1 indicates the frequencies CD4$^+$ cells among the Vimentin$^-$ αSMA$^+$ activated resident cardiac fibroblast population. The arrows from quadrant 3 indicate the frequencies CD4$^+$ cells among the Vimentin$^+$ αSMA$^-$ quiescent resident cardiac fibroblast population. The arrows from quadrant 2 indicate the frequencies of CD4$^+$ cells among the Vimentin$^+$ αSMA$^+$ population.

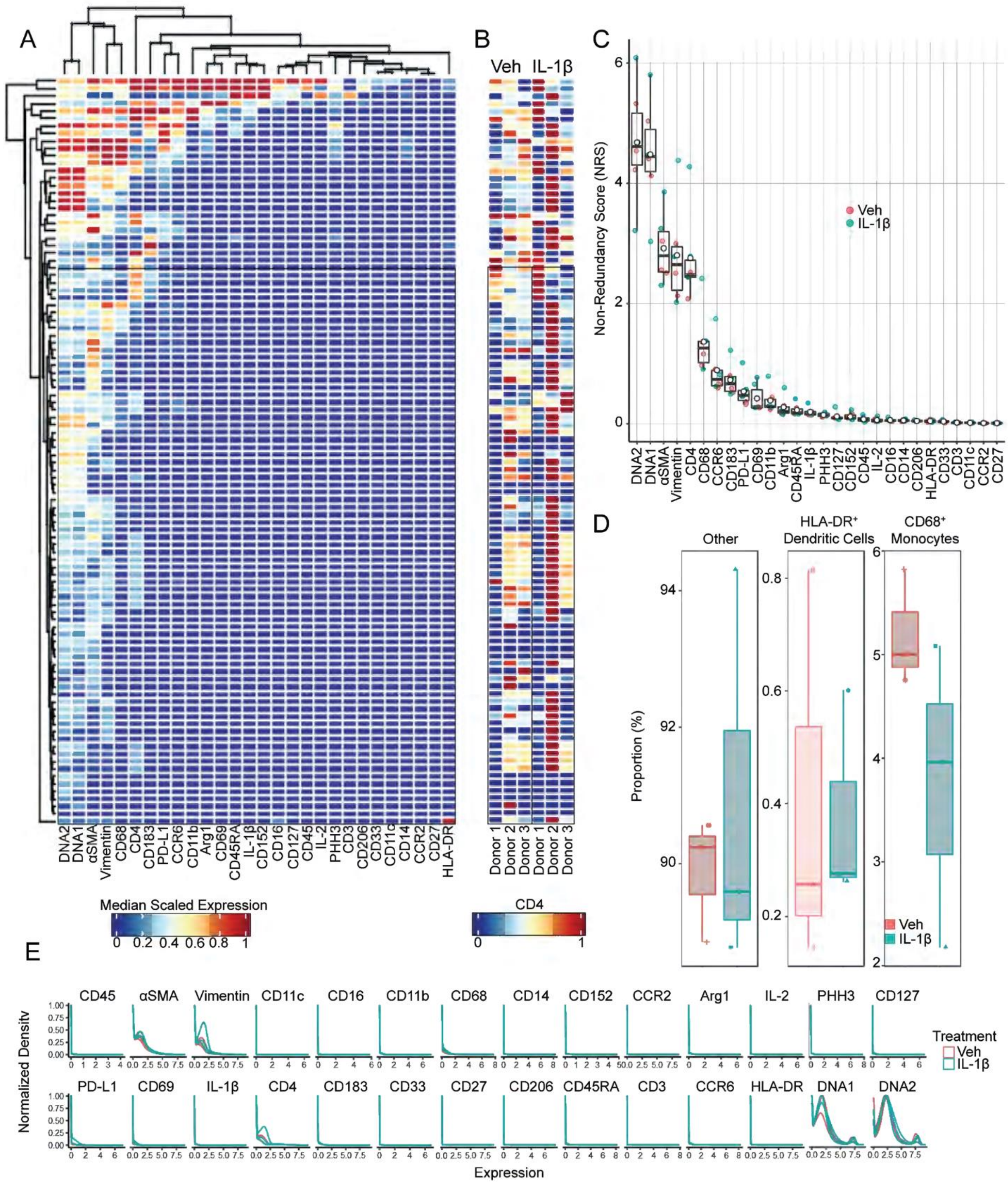

Figure S8: **IL-1β mediated expansion of CD4 expressing cells detected using single cell mass cytometry.** (A) Heatmap of marker intensities of all the 26 markers that were used in the mass cytometry analysis of resident primary hVCF. (B) Single cells expression of CD4 in Veh and IL-1β for all the 3 donors. (C) Quantification of all the markers in Veh and IL-1β represented as a non-redundancy score. (D) Quantification of the HLA-DR, and myeloid and other cell populations treated with Veh and IL-1β based on the heatmap. (E) Normalized density expression, based on the heatmap of the markers in Veh and IL-1β treated cells, which were measured using mass cytometry. N=3 biological replicates.

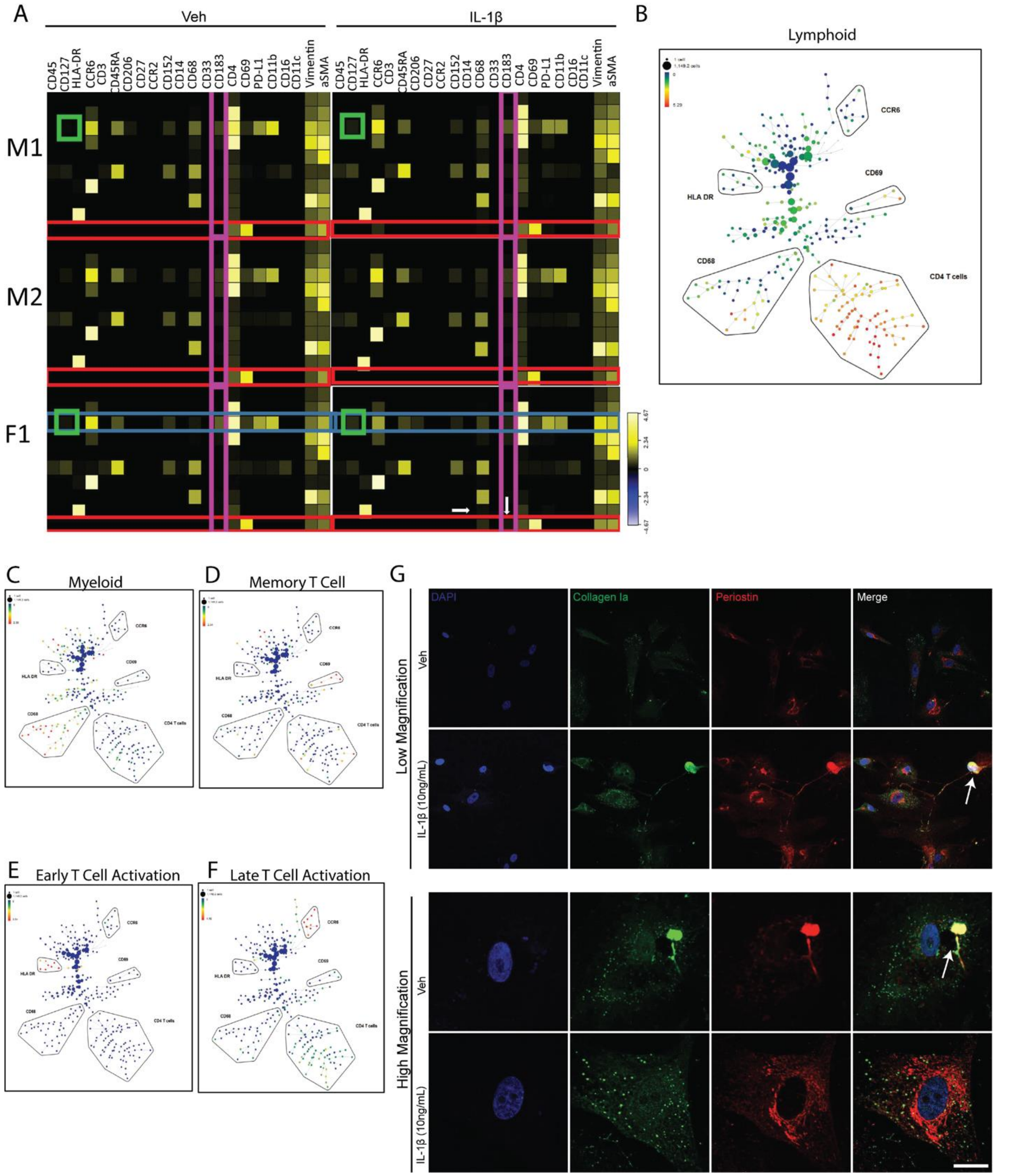

Figure S9: Delineation of cardiac fibroblast population sub-types using heatmaps and SPADE algorithms (A) Heat map representing the expression of all the markers tested under Veh and IL-1β treatment. Subtle changes identified qualitatively from the expression levels is marked by colored boxes (red, purple, and green). The expression of all the markers is on the X-axis. Based on the expression pattern of specific immune markers, the identity of the fibroblasts is determined. Since these cardiac fibroblast populations have not been characterized before, we have not marked the Y-axis. SPADE trees of (B) Lymphoid, (C) Myeloid (D) Memory T cell (E) Early T cell activation and (F) Late T cell activation identifying abundant populations and rare populations. The number of branches depends on the number of populations identified. A large tree with numerous branches represents highly heterogenous cell populations. Nodes represent the intensity of expression of markers. (G) Low and high magnification

images of Veh or 24 h IL-1β(10ng/mL) treated human cardiac fibroblast cells immunostained with Collagen 1α or Periostin. Bottom Panel: High magnification images of Veh or 24 h IL-1β(10ng/mL) treated human cardiac fibroblast cells immunostained with Collagen 1α or Periostin. Scale bars 20μM and 100μM.

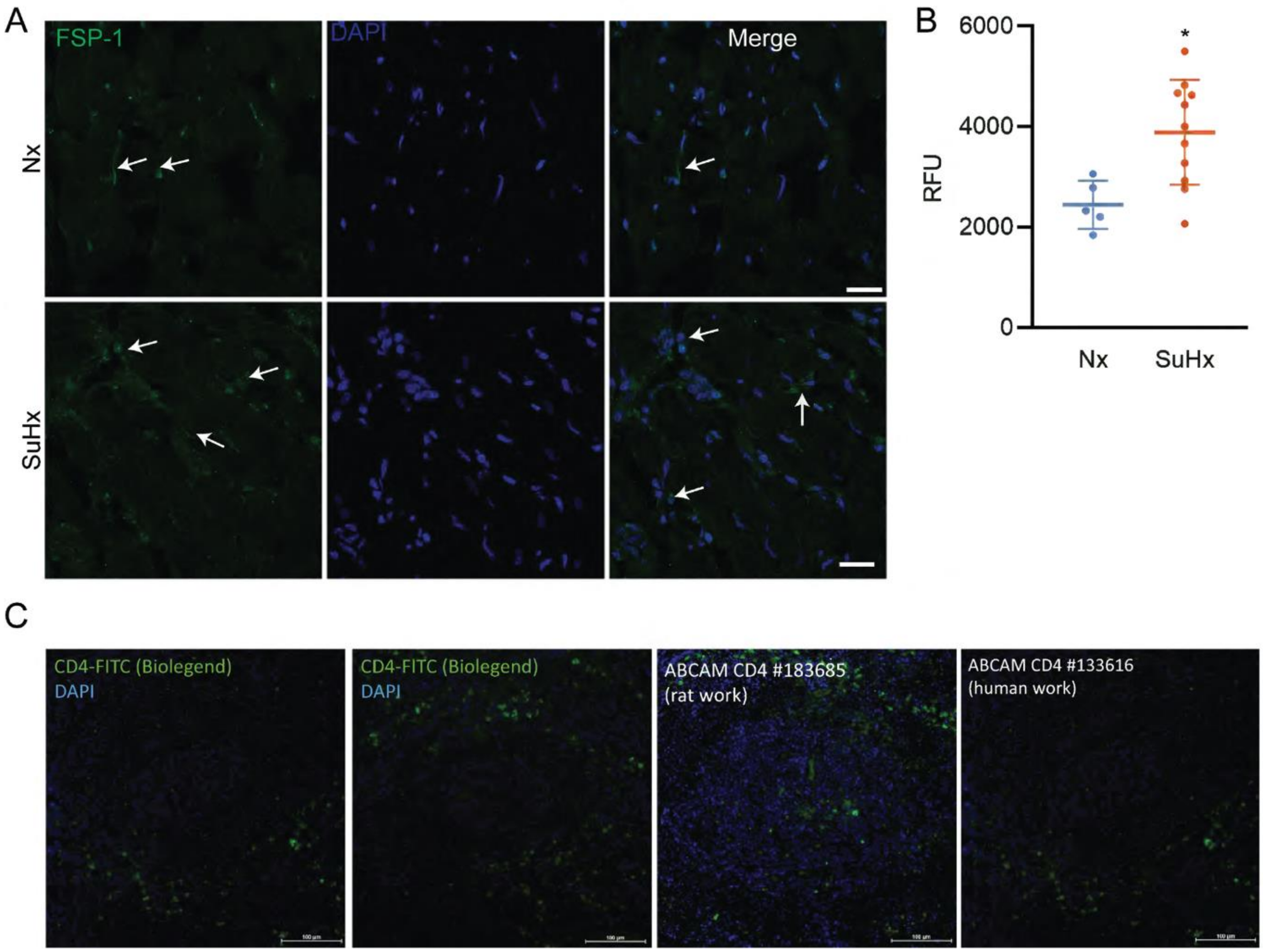

Figure S10: Characterization of SUGEN/hypoxia rat model of pulmonary hypertension. (A) Fibroblast specific protein (FSP-1) staining of cardiac fibroblasts in rat RVs of both Nx and SuHx groups (Figure S3). (B) Quantification of expression expressed in relative fluorescence unit (RFU). Values mean ± SD (n=10). The comparisons were made with unpaired t test. **P*<0.05 vs Nx rats. (C) The specificity of the CD4 antibodies were validated on OCT embedded rat spleen sections by immunostaining.

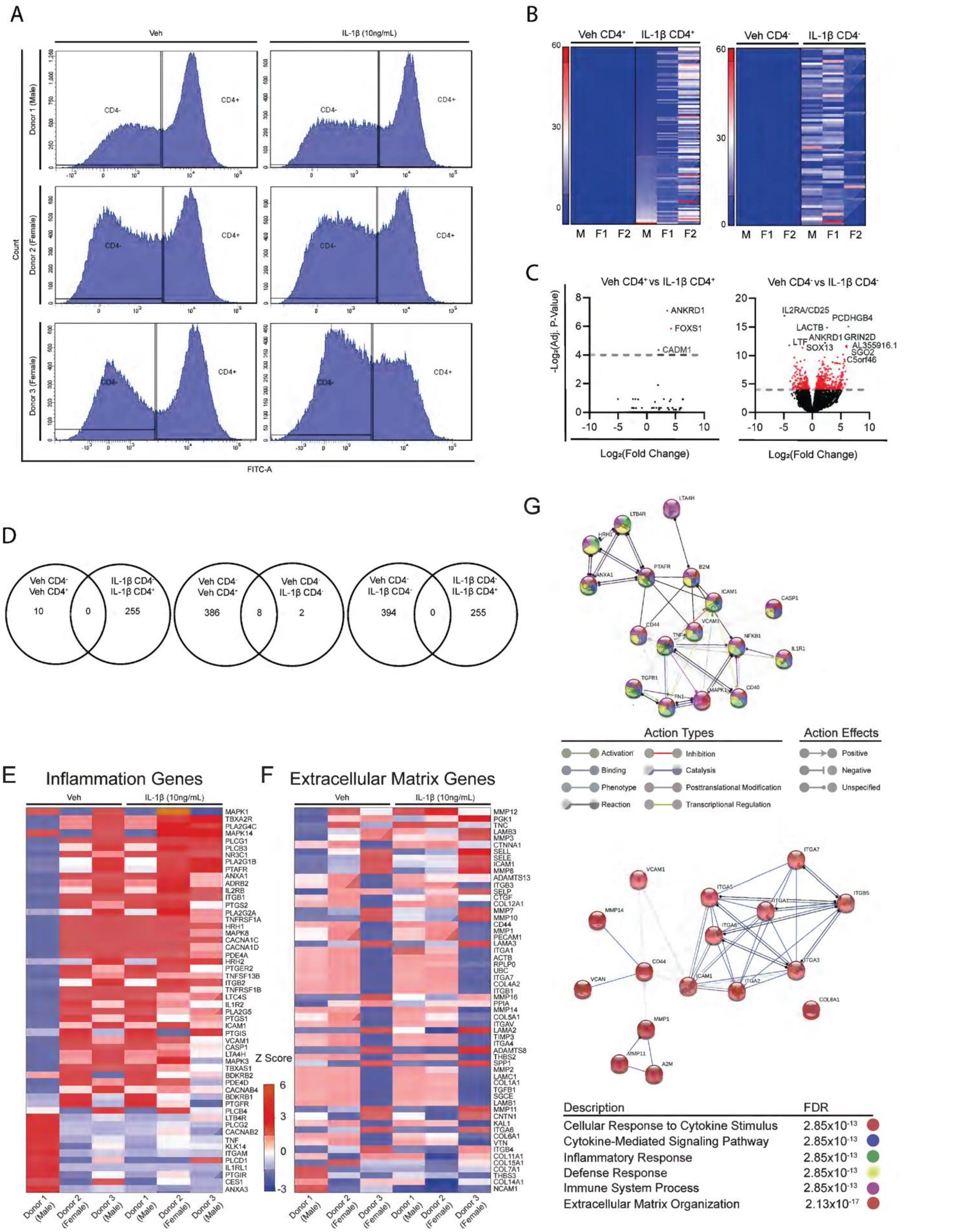

Figure S11: IL-1β mediated transcriptomic switch. (A) Graphical representation of untreated or IL-1β treated human ventricular cardiac fibroblast sorted into $CD4^+$ and $CD4^-$ population for RNA seq analysis for all the three human donor cells. (B) Heatmaps showing the expression pattern of genes uniquely identified in Veh CD4+, Veh CD4-, IL-1β CD4+ and IL-1β CD4- populations. (C) Volcano plot showing the number of significantly upregulated and downregulated genes for each of the comparisons. False

Discovery Rate (FDR) (adjusted *P* value <0.01). (D) Venn diagram showing the number of common genes among the Veh and IL-1β among the unique gene identified for all the populations. Heatmaps and Z-scores representing differential gene expression between vehicle- and IL-1β-treated cells affecting (E) inflammation and (F) extracellular matrix remodeling using R package (heatmap.2). Red denotes high expression, and blue denotes low expression. (G) String pathway analysis using CYTOSCAPE. Gene networks represent undirected weighted partial correlation network constructed as a force directed graph. The network nodes represent individual genes that are differentially expressed. Edges connect the modes, and edge weights are colored based on relative activity.

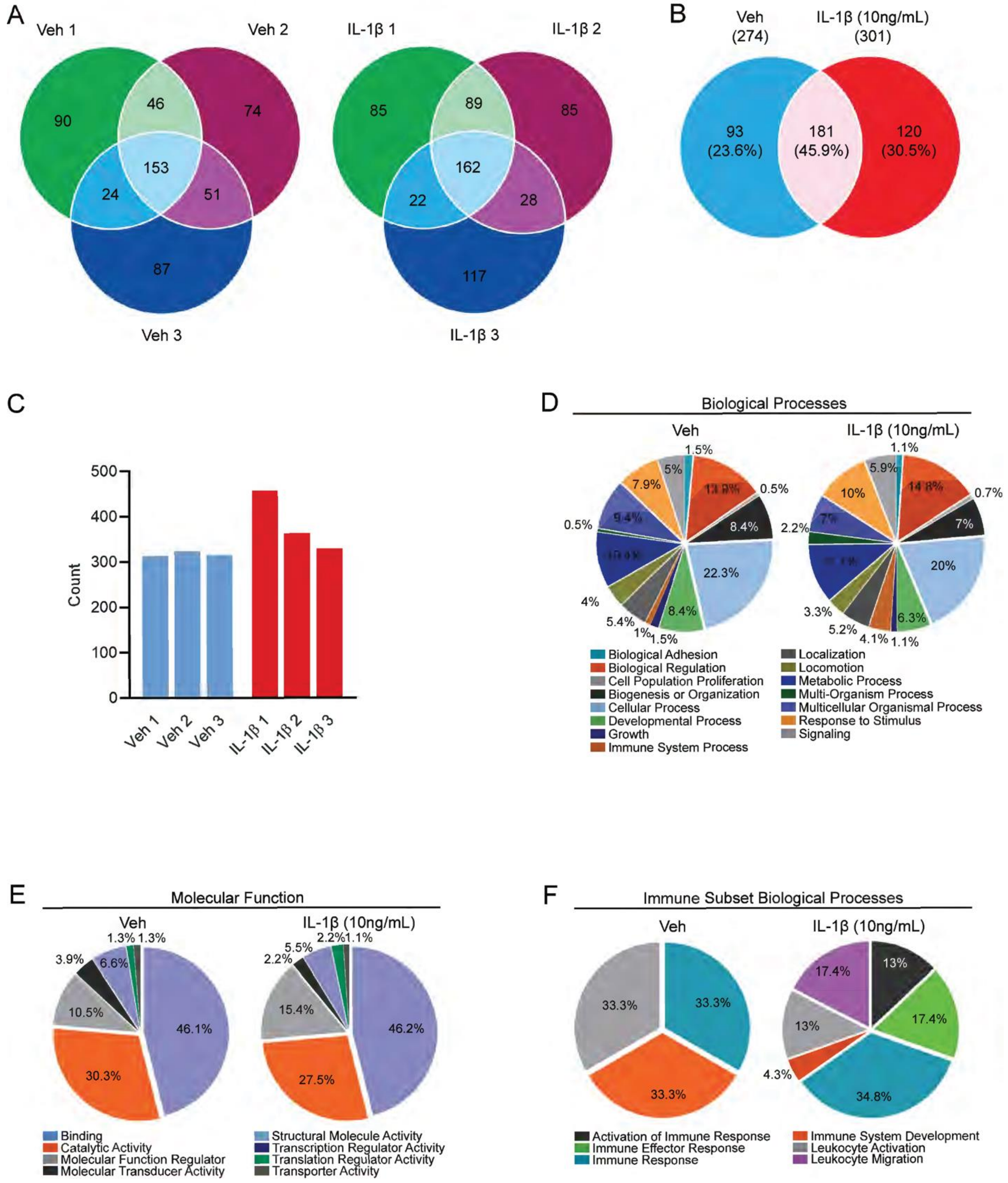

Figure S12: Non-immune cardiac fibroblasts secrete immunomodulatory proteins in response to IL-1β treatment. (A) Multivariable proteomic profile of proteins in technical replicates of cardiac fibroblasts (ID# 62122) conditioned media volumes (1 mL, 2 mL and 4 mL). Venn diagram represents proteins secreted in the conditioned media of primary human ventricular cardiac fibroblasts treated with Veh or 24 h IL-1β (10 ng/mL) from three samples determined using Venny v2.0. (B) Venn diagram represents

proteins that are unique to either the Veh or IL-1β or proteins common to both the conditions. (C) Average number of proteins detected in the conditioned media of cardiac fibroblasts treated with Veh or IL-1β for each of the replicate (D) Pathway enrichment analysis of the metabolites differentially expressed with IL-1β treatment in DAVID (https://david.ncifcrf.gov/). The length of the bar corresponds to significance of the pathway based on the –Log q value and the multiplicity of the components corrected using Benjamini-Hochberg. (E) Pie charts based on gene ontology showing the number and proportion of secreted proteins that belong to different biological processes in both Veh and IL-1β groups determine using PANTHER (http://www.pantherdb.org/) (F) Pie charts showing the number and proportion of secreted proteins that belong to different molecular function in both Veh and IL-1β groups. (G) Pie charts showing the number and proportion of secreted proteins that belong to different immune subsets in both Veh and IL-1β groups. Secretory protein belonging to the functional process, leukocyte migration and activation of immune response were distinctly upregulated with IL-1β.

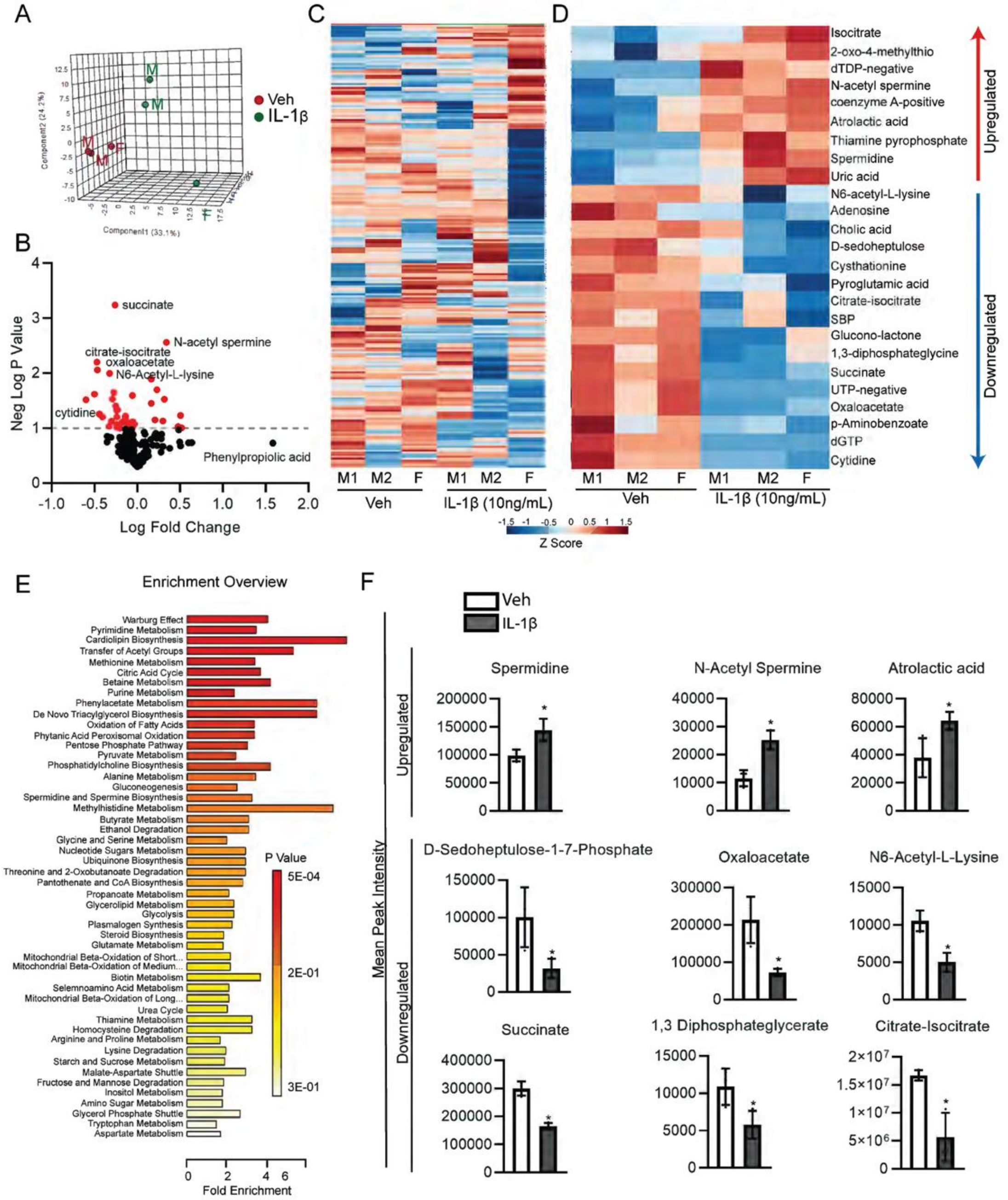

Figure S13: IL-1β modulates inflammatory and extracellular secretome in hVCF: (A) Cardiac fibroblasts were treated with IL-1β for 96h and principal component analysis of a cluster of metabolomic profiles relative to control. Multivariable metabolite profile of individuals (ID# 62122; male, ID#

1281202; male and ID#534282; female) was reduced to a single dot and represented based on consensus clustered alignment. Principal component 1 (PC1) on the x-axis versus principal component 2 (PC2) on the y-axis. (B) Volcano plot showing global expression of secreted metabolites in the conditioned media positively and negatively modulated upon treatment with IL-1R ng/mL for 24 hr. Each dot represents a differentially expressed metabolite. The horizontal line marks a P value of 0.01, and the red dots represent significant metabolites. (C) Heatmap columns comparing the expression of secreted metabolites identified by unsupervised hierarchical consensus clustering. Each row represents individual metabolites identified in the conditioned media of vehicle- and IL-1β-treated cells. The displayed color codes represent Z scores (SD above or below the mean); n=3 biological replicates. (D) Top 25 metabolites showing a significant expression of upregulated or downregulated metabolites (**P* <0.05) between Veh- and IL-1R-treated cell conditioned media) are represented as a heatmap; n=3 biological replicates. (E) Pathway enrichment analysis of the metabolites differentially expressed with IL-1β treatment. Length of the bar corresponds to fold enrichment, and the color suggests the significance of the pathway based on the *P* value. (F) Quantification of the metabolites is represented as Mean ± SD from 3 different human donors. (**P* <0.05, two-tailed *t* test, Mann Whitney post-hoc test).

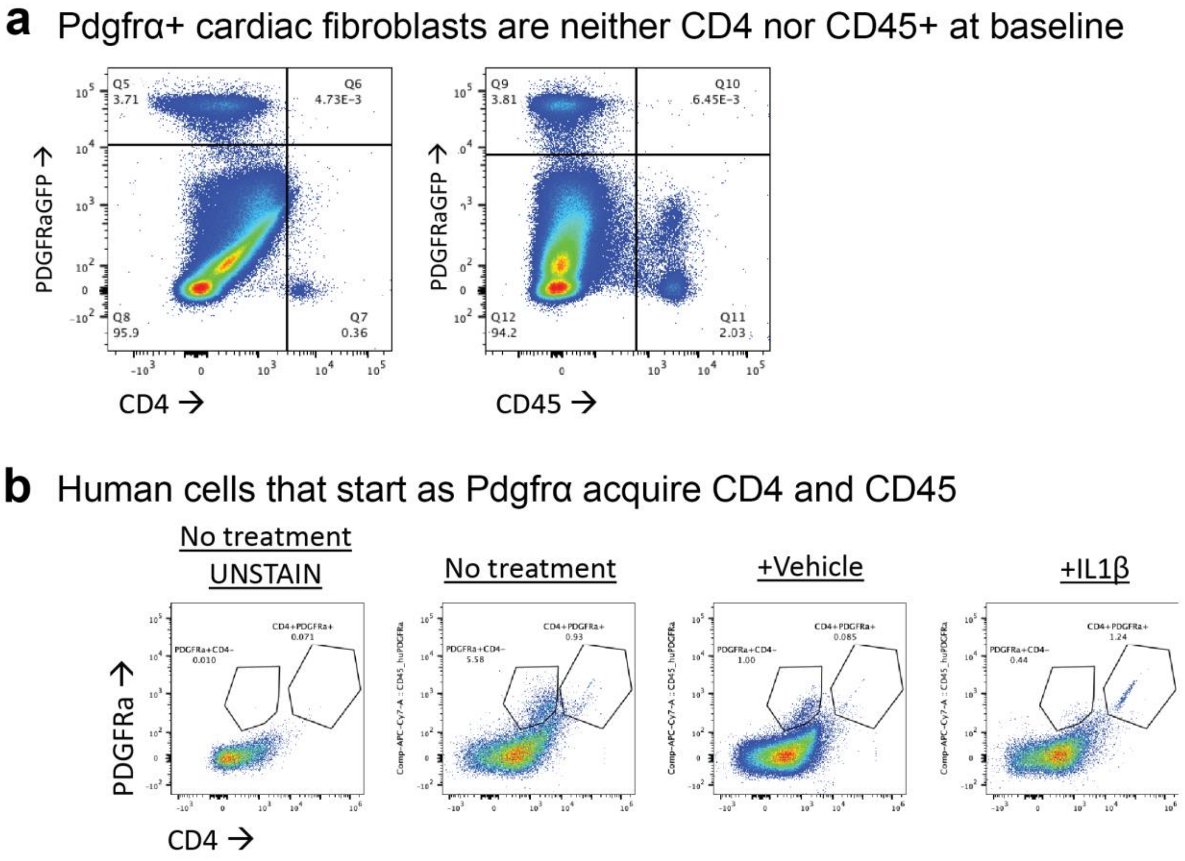

Figure S14: Mouse and Human Pdgfrα positive fibroblasts transition to CD4 positive. (A) Flow cytometric analyses of mouse cardiac interstitial cells isolated from the PdgfrαGFP reporter mouse line. Y-axis depicts the GFP fluorescent intensity versus on the X-axis fluorescent intensity for either CD4 (left plot) or CD45 (right plot) antibody labeling conjugated with fluorophores. As seen in the top right quadrant of each flow plot Pdgfrα positive fibroblast does not express either CD4 or CD45. **(B)** Flow cytometry analyses human fibroblasts for each treatment group as labeled. Y-axis depicts the fluorescent intensity for Pdgfrα antibody labeling versus on the X-axis fluorescent intensity for CD4 antibody labeling conjugated with fluorophores. Human cardiac fibroblasts that were originally Pdgfrα positive and CD4 negative are shown on the left pentagon shaped gate later acquired a Pdgfrα negative and CD4 positive phenotype shown on the right hexagon flow gates. Each experiment has a biological n=3 and technical replicates of n=3.

Supplementary Tables

Table S1: Overview of characteristics of donors of primary human ventricular cardiac fibroblasts

Human Donor #ID	Age (yr)	Sex	Isolation Location	Medications	Diabetes	Alcohol	Smoking
67771	67	F	Left Ventricle	Xanax, Prozac, Neurontin, Dilaudid, Meloxicam, Morphine, Zyprexa, Oxycodone, Flomax, Levophed, Vancomycin, Clindamycin	No	Yes	1 pack per day, 30+ years
62122	73	M	Left Ventricle	Not known	No	Yes	No
1281202	67	M	Left Ventricle	Neosynephrine, Solumedrol, Lasix, Thyroxine, Zosyn, Meropenem, Cleocin	No	No	No
534282	63	F	Left Ventricle	Not known	No	Yes	Yes
TL210281	73	M	Left Ventricle	Not known	No	Yes	1-2 packs per day

Table S2: Antibodies used for immunostaining and immunoblotting during *in vitro* and *in vivo* experiments.

Antibody (Ab)	Clone	Source	Identifier (Cat#)
α-actin smooth muscle actin (mouse)	1A4	Sigma-Aldrich	A2547
IL1 Receptor I/IL-1R-1 (rabbit)	N/A	Abcam	ab106278
β-actin (mouse)	AC-15	Sigma-Aldrich	A5441
Vinculin (rabbit)	N/A	Santa Cruz Biotechnology	sc-5573
Periostin (mouse)	5B2.3	Sigma-Aldrich	MABS1183
S100A4/FSP1 (recombinant)	EPR2761	Abcam	ab124805
PDGFR-β (mouse)	D-6	Santa Cruz Biotechnology	sc-374573
Collagen I (rabbit)	N/A	Abcam	ab34710
VE-cadherin (mouse)	F-8	Santa Cruz Biotechnology	sc-9989
CD4 (rabbit mAB)	EPR6855	Abcam	ab133616
CD68 (mouse)	KP1	Abcam	ab955
Vimentin (mouse)	AMF-17b	Developmental Studies Hybridoma Bank	AMF-17b
CD3-APC (rat)	REA223	Miltenyi Biotec	130-103-132
CD4 (Domain 1)-PE (rat)	REA482	Miltenyi Biotec	130-107-668
p-p38 MAPK (Thr180/Tyr182) (Rabbit mAB)	D3f9	Cell Signaling Technologies	4511S
p38 MAPK (rabbit mAB)	D13E1	Cell Signaling Technologies	8690S
p-p65NFκB (Ser 536) (rabbit mAB)	93H1	Cell Signaling Technologies	3033
NFκB p-65 (rabbit mAB)	D14E12	Cell Signaling Technologies	8242
CD4-FITC (mouse)	RMA-5	Biolegend	100510

Table S3: Heavy metal conjugated antibodies used for immunostaining and immunoblotting during *in vitro* and *in vivo* experiments.

Ab #		Target	Metal tag Ab (amu)	Marker functions	Ab Clone	Catalog number	Company
1		α-SMA	141Pr	Myofibroblast marker	1A4	3141017D	Fluidigm
2		Vimentin	143Nd	Cardiac fibroblast marker	D21H3	3143027D	Fluidigm
3		CD11c	147SM	Dendritic cell marker	Bu15	3147008B	Fluidigm
4		CD16	148Nd	Neutrophil	3G8	3148004B	Fluidigm
5		CD11b	149Sm	Macrophages and Microglia	EPR1344	3149028D	Fluidigm
6		PD-L1	150Nd	Expansion of CD4 and CD8-T cells	E1L3N	3150031D	Fluidigm
7		CD69	153Eu	Activation marker on hematopoietic stem cells	FN50	V00399	Longwood
8		IL-1β	154Sm	Adaptive Immunity	H1b-27	V05038	Longwood
9		CD4	155Gd	T-helper cell marker	RPA T4	V04286	Longwood
10		CD183	156Gd	Effector T-cells	G025H7)	3156004B	Fluidigm
11		CD33	158Gd	Myeloid marker	WM53	3158001B	Fluidigm
12		CD68	159Tb	Classical macrophage	KP1	3159035D	Fluidigm
13		CD14	160Gd	LPS receptor	M5E2	3160001B	Fluidigm
14		CTLA-4	161Dy	Activated T-lymphocyte	14D3	3161004B	Fluidigm
15		CCR2	163Dy	Resident macrophage	K036C2	V17774	Longwood
16		Arginase1	164Dy	Alternatively activated macrophages	3164027D	D4E3M	Fluidigm
17		IL-2	166Er	T-cell growth factor	MQ1-17H12	3166002B	Fluidigm
18		CD27	167Er	Co-stimulatory marker expressed on T-cell, B-cell and NK cell	O323	3167002B	Fluidigm
19		CD206	168Er	Alternatively activated macrophages	15-2	3168008B	Fluidigm
20		CD45RA	169Tm	Helper T-cell	HI100	3169008B	Fluidigm
21		CD3	170Er	CD3 T-cell co-receptor	UCHT1	3170001B	Fluidigm
22		CCR6	171Yb	Functional marker for Th17 cell	G034E3	353427	Biolegend
23		PHH3	175Lu	Cell proliferation marker	3176023D	HTA28	Fluidigm
24		CD127/IL-7RA	176Yb	Treg cell marker	A019D5	3176004B	Fluidigm
25		HLA-DR	174Yb	Cytotoxic-T-cell activation	L243	3174001B	Fluidigm
26		CD45	89Y	Pan immune cell marker	HI30	3089003B	Fluidigm

αSMA: alpha smooth muscle actin; IL-1β: Interleukin 1 beta; HLA-DR: human leukocyte antigen-DR; PD-L1: Programmed death ligand 1

Table S4. Baseline characteristics of patient donors of right ventricular tissue

Patient ID#	Age at Death (yr)	Sex	Cause of Death	Final Anatomic Diagnosis
A	63	M	Progressive supranuclear palsy, leading to dysphagia, complicated by aspiration pneumonia	Not available
B	66	M	Atherosclerotic and hypertrophic cardiovascular disease	Echocardiography showed dilated right ventricle. At autopsy, there were many signs of atherosclerotic and hypertensive changes. Atherosclerotic and hypertrophic cardiovascular disease, biventricular cardiac hypertrophy (500 g), remote myocardial infarct, posterior papillary muscle ,coronary artery atherosclerosis, left main coronary artery at origin: 50-75% occluded left anterior descending artery: 75-100% occluded, left circumflex artery: 50% occluded, right coronary artery: 50% occluded pulmonary artery atherosclerosis (consistent with sustained pulmonary hypertension) right ventricle dilatation, arterio-nephrosclerosis
C	61	F	Metastatic dedifferentiated endometroid adenocarcinoma of ovary complicated by intraperitoneal hemorrhage from liver biopsy	Not available
D	92	M	Cardiac amyloidosis leading to restrictive cardiomyopathy complicated by atherosclerotic and hypertensive cardiovascular disease	Amyloid cardiomyopathy, multifocal cardiac amyloid deposition confirmed with Congo red stain, atherosclerotic and hypertrophic cardiovascular disease (clinical history of systemic and pulmonary hypertension), cardiomegaly (500 gm) with biventricular hypertrophy coronary artery atherosclerosis, moderate: left anterior descending artery, 20-40% stenosis right coronary artery, 40% stenosis, subendocardial infarct, remote, posterior papillary muscle, left ventricle
E	32	M	Acute asthmatic attack	Not available
F	65	M	Primary pulmonary hypertension, leading to right ventricular dilation and hypertrophy, leading to right heart failure	primary pulmonary hypertension, cardiomegaly (890 gm), with right ventricular dilation and biventricular hypertrophy, thickening and enlargement of the tricuspid valve, arterio- and arteriolo-nephrosclerosis, bilateral kidneys, focal segmental glomerulosclerosis, bilateral kidneys, cystic emphysematous blebs, bilateral lungs, petechial hemorrhage, diffuse, stomach, multiple cortical cysts (up to 2.5 cm), bilateral kidneys atherosclerosis, thoracic and abdominal aorta

Table S5: Primer sequence of genes amplified to determine the gene expression levels in vehicle and IL1β treated rat cardiac fibroblast cells.

	**Forward Primer**	**Reverse Primer**
**Rat**		
Col-1	5'-TGCCGTGACCTCAAGATGTG-3′	5'-CACAAGCGTGCTGTAGGTGA-3′
Col-III	5'-ATGGTGGCTTTCAGTTCAGC-3′	5'-TGGGGTTTCAGAGAGTTTGG-3′
MMP-9	5'-GTACAGCCTGTTTCTGGTGGC-3'	5'-GGCCTTGGGTCAGGTTTAGAG-3'
MyD88	5'-TTCTCCAACGCTGTCCTGTC-3'	5'-AACTGAGATGTGTGCCCAGG-3'
IL-6	5'-TTGTTGACAGCCACTGCCTTCCC-3'	5'-TCTGACAGTGCATCATCGCTGTTCA-3'
CCL2	5’-GCCCCACTCACCTGCTGCTAC-3’	5’-GGTTCTGATCTCATTTGGTTCCG-3’
CCR2	5'-ATCCACGGCATACTATCAACATCTC-3’	5’-GACAAGGCTCACCATCATCGTAG-3'
IL1R	5’-CTT GCC GCA CGT CCT ACA CAT ACC-3’	5’-CGG GGA AGA AAA TCA GAG CAG GAG-3’
**Housekeeping genes**		
GAPDH	5'-CGCTAACATCAAATGGGGTG-3′	5'-TTGCTGACAATCTTGAGGGAG-3′
18sRNA	5’-AAACGGCTACCACATCCA-3’	5’-CTCATTCCAATTACAGGG-3’
**Human**		
IL-2	5'-CAT TGC ACT AAG TCT TGC ACT TGT CA-3'	5'-CGT TGA TAT TGC TGA TTA AGT CCC TG-3'
IL-6	5'-ATGAACTCCTTCTCCACAAGCGC-3'	5'-GAAGAGCCCTCAGGCTGGACTG-3'
IL-8	5'-TGACTTCCAAGCTGGCCGTGGCT-3'	5'-TCTCAGCCCTCTTCAAAAACTTCTC-3'
IL-10	5'-AAGCTGAGAACCAAGACCCAGACATCAAGGCG-3'	5'-AGCTATCCCAGAGCCCCAGATCCGATTTTGG-3'
ICAM 1	5'-GAT TGT CAT CAT CAC TGT GGT AG-3'	5'-GCC TGT TGT AGT CTG TAT TTC TT-3'
CD4	5'- GGA GTC CCT TTT AGG CAC TTG C -3'	5'-AAGACAGTGCATGTCCAGGTG-3'
TGF-B	5'-GGCCCTGCCCCTACATTT-3'	5'-CCGGGTTATGCTGGTTGTACA-3'
**Housekeeping genes**		
β-Actin	5’-CTTTCGTGTAAATTATGTAATGCA-3’	5’-TACATCTCAAGTTGGGGGA-3’
GAPDH	5'-ACCACAGTCCATGCCATCAC-3'	5'-TCCACCACCCTGTTGCTGTA-3'

Table S6: Differentially regulated extracellular matrix genes in the Veh and IL-1β (10ng/mL) treated hVCF using taqman gene array.

Gene	Gene name	2^-ΔCt (Veh)	2^-ΔCt (IL-1B)	Standard deviation (Veh)	Standard deviation (IL-1B)	Fold Change (IL-1B/Veh)	P value	(-logP)	FDR q Value
NCAM1	neural cell adhesion molecule 1	0.75	0.41	1.16	0.14	0.55	0.17	0.78	10.00
TNC	tenascin C	4.47	10.87	2.95	9.20	2.43	0.25	0.60	7.53
FN1	fibronectin 1	20.34	40.36	20.26	0.00	1.98	0.28	0.56	5.54
COL12A1	collagen type XII alpha 1 chain	2.44	6.71	2.50	5.77	2.75	0.31	0.51	4.64
CD44	CD44 molecule	3.29	8.79	3.32	7.59	2.67	0.32	0.50	3.79
MMP12	matrix metallopeptidase 12	4.28	48.63	3.03	70.22	11.36	0.32	0.49	3.24
THBS3	thrombospondin 3	72.39	0.25	41.93	0.24	0.00	0.38	0.43	3.21
COL7A1	collagen type VII α1chain	4.34	0.41	2.89	0.14	0.10	0.38	0.42	2.85
PGK1	phosphoglycerate kinase 1	0.76	1.68	1.17	0.58	2.21	0.41	0.39	2.73
MMP14	matrix metallopeptidase 14	2.25	5.39	2.67	4.65	2.39	0.41	0.38	2.48
ITGB3	integrin subunit β 3	2.53	5.01	2.12	4.94	1.98	0.42	0.38	2.28
ACTB	actin β	1.03	1.98	0.83	1.98	1.93	0.42	0.38	2.10
ITGB2	integrin subunit β2	7.89	0.27	5.67	0.13	0.03	0.44	0.36	2.01
CTNNA1	catenin α1	1.08	2.59	1.33	2.34	2.41	0.44	0.35	1.89
COL14A1	collagen type XIV α1 chain	0.70	0.75	1.22	0.77	1.06	0.48	0.32	1.91
COL4A2	collagen type IV α2 chain	8.03	16.16	8.02	16.12	2.01	0.48	0.32	1.79
UBC	ubiquitin C	4.03	8.17	4.05	8.17	2.02	0.48	0.32	1.69
ITGA1	integrin subunit α1	1.98	4.11	2.07	4.08	2.07	0.48	0.32	1.60
THBS2	thrombospondin 2	4.02	8.08	4.02	8.12	2.01	0.48	0.32	1.52
MMP7	matrix metallopeptidase 7	0.06	13.29	0.12	22.20	235.17	0.48	0.31	1.45
ITGB4	integrin β4	2.61	0.81	1.96	0.37	0.31	0.49	0.31	1.41
ITGA6	integrin α6	4.51	1.01	2.99	0.86	0.22	0.50	0.30	1.35
CNTN1	contactin 1	37.21	5.63	21.99	8.88	0.15	0.50	0.30	1.30
COL15A1	collagen type XV α1 chain	0.78	0.84	1.17	0.30	1.08	0.53	0.28	1.32
MMP8	matrix metallopeptidase 8	0.13	0.31	0.16	0.26	2.37	0.53	0.28	1.27

ITGA7	integrin α7	8.46	13.50	6.76	16.86	1.60	0.57	0.24	1.31
MMP1	matrix metallopeptidase 1	2.01	3.41	1.76	4.17	1.69	0.57	0.24	1.27
PECAM1	platelet and endothelial cell adhesion molecule 1	1.98	3.35	1.74	4.07	1.69	0.57	0.24	1.23
SELL	selectin L	1.93	3.10	1.73	4.08	1.60	0.63	0.20	1.29
COL1A1	collagen type I α1 chain	11.33	19.72	13.09	19.88	1.74	0.64	0.19	1.28
MMP2	matrix metallopeptidase 2	1.83	3.28	2.21	3.25	1.79	0.65	0.19	1.25
ADAMTS 13	ADAM metallopeptidase with thrombospondin type 1 motif 13	0.78	1.52	1.18	0.88	1.94	0.65	0.19	1.22
MMP11	matrix metallopeptidase 11	7.37	2.36	4.56	3.55	0.32	0.67	0.18	1.21
SPP1	secreted phosphoprotein 1	2.63	1.24	1.99	1.05	0.47	0.67	0.17	1.19
SELE	selectin E	119.05	34.97	68.81	59.14	0.29	0.68	0.17	1.17
MMP10	matrix metallopeptidase 10	15.01	4.72	8.87	7.09	0.31	0.69	0.16	1.15
ICAM1	intercellular adhesion molecule 1	11.32	3.90	6.76	5.17	0.34	0.71	0.15	1.15
LAMA3	laminin α3	4.47	1.86	2.96	1.90	0.42	0.71	0.15	1.13
RPLP0	ribosomal protein lateral stalk subunit P0	3.30	4.42	3.32	3.78	1.34	0.72	0.14	1.11
TGFB1	transforming growth factor β1	12.41	16.42	12.31	14.22	1.32	0.73	0.14	1.09
LAMC1	laminin subunit γ1	8.07	10.68	8.03	9.24	1.32	0.73	0.14	1.06
SGCE	sarcoglycan epsilon	5.03	6.67	5.02	5.77	1.33	0.73	0.14	1.04
ITGAV	integrin subunit αV	1.98	2.72	2.07	2.30	1.38	0.73	0.14	1.02
ITGB1	integrin β1	8.15	10.74	8.14	9.30	1.32	0.73	0.13	1.00
KAL1	anosmin1	1.56	2.21	1.72	1.87	1.42	0.74	0.13	0.99
COL5A1	collagen type V α1 chain	0.77	1.41	1.17	1.01	1.83	0.75	0.12	0.98
MMP3	matrix metallopeptidase 3	18.20	7.31	11.03	7.49	0.40	0.76	0.12	0.97
VTN	vitronectin	1.08	1.66	1.33	1.37	1.53	0.76	0.12	0.95
MMP16	matrix metallopeptidase 16	1.10	1.04	1.25	0.36	0.94	0.76	0.12	0.94
LAMA2	laminin subunit α2	1.08	1.02	1.23	0.36	0.95	0.76	0.12	0.92
COL11A1	collagen type XI α1 chain	1.12	1.14	1.37	1.19	1.02	0.82	0.08	0.97

TIMP3	TIMP metallopeptidase inhibitor 3	10.37	9.89	8.26	9.87	0.95	0.85	0.07	0.98
ITGA4	integrin α4	5.24	5.02	4.20	4.98	0.96	0.85	0.07	0.97
LAMB3	Laminin β3	2.58	2.05	1.95	0.71	0.80	0.95	0.02	1.05
SELP	selectin P	1.73	2.15	2.09	1.53	1.25	0.96	0.02	1.05
COL6A1	collagen type VI α1 chain	1.00	1.36	1.40	1.07	1.37	0.97	0.01	1.04
CTGF	connective tissue growth factor	3.36	2.87	2.75	3.25	0.85	0.97	0.01	1.02
ADAMTS 8	ADAM metallopeptidase with thrombospondin type 1 motif 8	3.51	2.39	2.43	3.44	0.68	0.99	0.00	1.02
LAMB1	laminin β1	2.91	3.37	3.40	2.90	1.16	0.99	0.00	1.01
PPIA	peptidylprolyl isomerase A	0.79	1.17	1.18	0.77	1.49	1.00	0.00	1.00

Table S7: Summary of differentially regulated inflammation genes in the Veh and IL-1β (10ng/mL) treated hVCF using taqman gene array

Gene	Gene name	2^-ΔCt (Veh)	2^-ΔCt (IL-1B)	Standard deviation (Veh)	Standard deviation (IL-1B)	Fold Change (IL-1B/Veh)	P value	(-logP)	FDR q Value
ITGAM	integrin α M	13.70	98.40	22.00	65.46	7.18	0.10	1.00	6.35
PLCG2	phospholipase C gamma 2	1.07	1.08	0.47	0.46	1.01	0.12	0.91	3.85
PTGS2	prostaglandin-endoperoxide synthase 2	0.98	1.84	0.01	1.91	1.87	0.18	0.75	3.76
ANXA1	annexin A1	10.65	24.32	9.22	13.96	2.28	0.23	0.64	3.63
BDKRB2	bradykinin receptor B2	1.07	22.67	0.46	27.02	21.13	0.24	0.62	3.01
PLA2G4C	phospholipase A2 group IVC	1.04	1.25	0.35	1.08	1.19	0.26	0.59	2.69
PTGIS	prostaglandin I2 synthase	1.33	2.57	1.08	2.31	1.93	0.28	0.55	2.53
ANXA5	annexin A5	1.19	0.59	0.78	0.39	0.50	0.31	0.52	2.41
NR3C1	nuclear receptor subfamily 3 group C member 1	1.02	1.81	0.45	1.19	1.77	0.34	0.46	2.41
PLCG1	phospholipase Cγ1	1.01	1.69	0.02	0.60	1.67	0.35	0.46	2.20
PLCE1	phospholipase C€1	1.58	0.76	1.74	0.62	0.48	0.36	0.45	2.05
VCAM1	vascular cell adhesion molecule 1	1.09	2.44	0.39	2.51	2.24	0.36	0.45	1.88
ITGB1	integrin β1	1.00	1.34	0.00	0.58	1.34	0.36	0.44	1.76
BDKRB1	bradykinin receptor β1	1.18	22.84	0.78	36.92	19.32	0.37	0.43	1.65
PLCB4	phospholipase C β4	1.42	1.76	1.02	2.01	1.24	0.40	0.40	1.68
IL1R2	interleukin 1 receptor type 2	1.04	1.68	0.37	0.75	1.61	0.41	0.39	1.61
PDE4B	Phosphodiester-ase β4	1.04	1.94	0.36	2.69	1.86	0.41	0.39	1.53
CACNAB4	calcium channels	1.42	0.64	1.47	0.22	0.45	0.42	0.38	1.47
KLKB1	kallikrein β1	16.40	9.42	16.42	6.16	0.57	0.43	0.37	1.42
CES1	Carbonxyesterase 1	2.11	0.83	2.53	0.38	0.39	0.44	0.36	1.37
TNFRSF1 B	TNF receptor superfamily member 1β	2.11	8.22	2.49	9.89	3.89	0.44	0.36	1.31

PTGS1	prostaglandin-endoperoxide synthase 1	1.51	7.03	1.56	5.67	4.67	0.45	0.35	1.28
A2M	α2 macroglobulin	1.67	3.34	1.50	3.18	2.00	0.46	0.34	1.25
LTB4R	leukotriene β4 receptor	1.16	3.18	0.77	4.21	2.73	0.46	0.34	1.21
TBXA2R	thromboxane A2 receptor	1.04	1.15	0.37	1.18	1.11	0.48	0.32	1.21
HRH1	histamine receptor H1	1.64	2.47	1.44	1.43	1.51	0.52	0.29	1.25
MAPK14	mitogen-activated protein kinase14	1.03	1.45	0.36	0.97	1.41	0.52	0.29	1.21
PTGER2	prostaglandin E receptor 2	1.09	1.51	0.46	1.54	1.38	0.52	0.28	1.18
PLA2G5	phospholipase A2 group V	1.17	1.18	0.78	0.79	1.01	0.53	0.28	1.15
LTA4H	leukotriene A4 hydrolase	1.40	1.41	1.00	1.01	1.01	0.53	0.28	1.11
IL1R1	interleukin 1 receptor type 1	3.08	6.75	4.38	8.13	2.19	0.53	0.28	1.08
CASP1	caspase 1	2.04	3.72	1.92	3.80	1.83	0.53	0.27	1.05
IL2RG	interleukin 2 receptor subunit γ	0.05	0.03	0.07	0.02	0.55	0.57	0.24	1.09
PDE4D	Phosphodiester-ase 4D	1.33	2.08	1.07	2.53	1.56	0.60	0.22	1.11
ICAM1	intercellular adhesion mol 1	13.71	6.56	22.68	2.84	0.48	0.62	0.21	1.11
PTAFR	platelet activating factor receptor	1.69	2.16	1.51	0.93	1.28	0.62	0.21	1.09
PDE4A	Phosphodiester-ase 4A	1.17	5.84	0.76	8.82	5.01	0.64	0.19	1.09
CACNA1C	calcium voltage-gated channel subunit α1 C	1.17	1.51	0.78	0.88	1.29	0.64	0.19	1.07
NFKB1	nuclear factor kappa β1	1.19	1.54	0.79	0.89	1.29	0.65	0.19	1.05
TNFSF13 B	tumor necrosis factor superfamily member 13b	1.69	2.60	1.55	3.46	1.54	0.66	0.18	1.03
ITGB2	integrin subunit β2	1.76	1.17	2.00	0.78	0.67	0.66	0.18	1.01
PLA2G1B	phospholipase A2 group IB	0.04	0.07	0.05	0.05	1.65	0.66	0.18	0.99

PTGDR	prostaglandin D2 receptor	1.04	2.25	0.36	2.98	2.16	0.67	0.17	0.98
CD40	CD40 molecule	1.69	2.16	1.52	0.92	1.28	0.67	0.17	0.96
IL1RAPL2	interleukin 1 receptor accessory protein like 2	1.00	1.35	0.01	0.58	1.35	0.67	0.17	0.94
PTGFR	prostaglandin F receptor	1.17	1.00	0.77	0.01	0.86	0.68	0.17	0.93
CYSLTR1	cysteinyl leukotriene receptor 1	1.74	1.31	1.59	0.43	0.75	0.70	0.16	0.94
PTGIR	prostaglandin I2 (prostacyclin) receptor (IP)	1.33	3.40	1.07	2.68	2.56	0.73	0.14	0.95
IL1RL1	interleukin 1 receptor like 1	1.19	1.79	0.77	2.03	1.51	0.77	0.11	0.99
PLCD1	phospholipase C delta 1	1.08	3.01	0.47	3.12	2.79	0.81	0.09	1.02
TNF	tumor necrosis factor	1.07	1.08	0.48	0.48	1.01	0.86	0.07	1.06
TBXAS1	thromboxane A synthase 1	1.68	1.89	1.50	1.25	1.13	0.88	0.05	1.07
ADRB2	Adrenoceptor β2	1.04	1.14	0.36	1.17	1.10	0.89	0.05	1.06
MC2R	melanocortin 2 receptor	1.17	1.50	0.78	0.87	1.28	0.91	0.04	1.06
ANXA3	annexin A3	1.97	1.78	2.61	1.27	0.90	0.91	0.04	1.05
CACNAB2	calcium channels	1.03	1.01	0.46	0.44	0.98	0.97	0.02	1.09
MAPK3	mitogen-activated protein kinase 3	1.09	1.10	0.48	0.49	1.00	0.98	0.01	1.08
IL2BR	interleukin 2 receptor β	1.26	1.28	1.07	0.49	1.01	0.98	0.01	1.07
TNFRSF1 A	TNF receptor superfamily member 1A	4.81	11.85	6.95	12.29	2.46	0.98	0.01	1.05
PLCB3	phospholipase C β3	2.79	0.89	3.40	0.72	0.32	0.99	0.01	1.04
LTC4S	leukotriene C4 synthase	1.77	1.97	1.65	2.17	1.11	0.99	0.00	1.02
CACNA1 D1	calcium channels	1.01	1.01	0.01	0.88	1.01	0.99	0.00	1.01
MAPK8	mitogen-activated protein kinase 8	1.78	3.24	2.02	4.31	1.82	0.99	0.00	0.99

Table S8: Biological process gene ontology (GO:BP) and cellular component gene ontology (GO:CC), obtained through GProfiler, of secreted proteins expressed in conditional media of human ventricular cardiac fibroblasts treated with IL-1β (10ng/mL). Ontology terms represented with P value of 0.001 or less.

GO:BP

**Term Name**	**Term ID**	P_{adj}
Humoral Immune Response	GO:006959	1.446×10^{-13}
Immune Effector Process	GO:0002252	1.838×10^{-13}
Leukocyte Mediated Immunity	GO:0002443	2.231×10^{-12}
Vesicle-Mediated Transport	GO:0016192	2.910×10^{-11}
Immune Response	GO:0006955	4.228×10^{-11}
Immune System Process	GO:0002376	7.294×10^{-11}
Platelet Degranulation	GO:0002576	1.149×10^{-10}
Neutrophil-Mediated Immunity	GO:0002446	6.557×10^{-6}
Neutrophil Activation	GO:0042119	6.557×10^{-6}
Acute-Phase Response	GO:0006953	6.716×10^{-5}
Lymphocyte-Mediated Immunity	GO:0002449	4.715×10^{-5}

GO:CC

**Term Name**	**Term ID**	P_{adj}
Extracellular Exosome	GO:0070062	7.413×10^{-22}
Collagen-Containing Extracellular Matrix	GO:0062023	1.948×10^{-17}
Extracellular Matrix	GO:0031012	9.520×10^{-15}
Secretory Granule Lumen	GO:0034774	3.445×10^{-12}
Cytoplasmic Vesicle Lumen	GO:0060205	4.274×10^{-12}

Table S9: Biological process gene ontology (GO:BP) level 3, immune subset identified through Database for Annotation, Visualization, and Integrated Discovery (DAVID) v6.8 of secreted proteins expressed uniquely in conditional media of human ventricular cardiac fibroblasts treated with IL-1β (10ng/mL). Multiplicity is controlled through Benjamini-Hochberg test.

Term	Gene Name	%	P Value	Fold Enrichment	Benjamini-Hochberg
GO:0006952~defense response	complement factor H(CFH), amyloid P component, serum(APCS), attractin(ATRN), complement C1q C chain(C1QC), C-C motif chemokine ligand 2(CCL2), orosomucoid 1(ORM1), serpin family A member 3(SERPINA3), orosomucoid 2(ORM2), apolipoprotein A1(APOA1), complement C4A (Rodgers blood group)(C4A), apolipoprotein A2(APOA2), serpin family A member 1(SERPINA1), complement component 4 binding protein alpha(C4BPA), peptidoglycan recognition protein 2(PGLYRP2), complement C5(C5), complement C6(C6), CD5 molecule like(CD5L), histone cluster 2 H2B family member e(HIST2H2BE), interleukin 1 beta(IL1B), C-X-C motif chemokine ligand 5(CXCL5), C-X-C motif chemokine ligand 1(CXCL1), haptoglobin(HP), integrin subunit beta 1(ITGB1), kallikrein B1(KLKB1), low density lipoprotein receptor(LDLR), immunoglobulin heavy constant gamma 4 (G4m marker)(IGHG4), interleukin 6(IL6), kininogen 1(KNG1), inter-alpha-trypsin inhibitor heavy chain family member 4(ITIH4), complement factor B(CFB), intercellular adhesion molecule 1(ICAM1), joining chain of multimeric IgA and IgM(JCHAIN), serpin family F member 1(SERPINF1), C-X-C motif chemokine ligand 8(CXCL8), hemopexin(HPX)	38.88889	6.12E-14	4.316775	3.76E-11
GO:0006959~humoral immune response	immunoglobulin kappa variable 1D-33(IGKV1D-33), complement factor H(CFH), complement C1q C chain(C1QC), C-C motif chemokine ligand 2(CCL2), bone marrow stromal cell antigen 1(BST1), immunoglobulin kappa variable 4-1(IGKV4-1), immunoglobulin heavy constant gamma 4 (G4m marker)(IGHG4), complement C4A (Rodgers blood group)(C4A), immunoglobulin kappa variable 3-20(IGKV3-20), interleukin 6(IL6), complement component 4 binding protein alpha(C4BPA), complement factor B(CFB), complement C5(C5), joining chain of multimeric IgA and IgM(JCHAIN), complement C6(C6), histone cluster 2 H2B family member e(HIST2H2BE), hemopexin(HPX)	18.88889	1.99E-13	12.83395	6.12E-11
GO:0006956~complement activation	immunoglobulin kappa variable 1D-33(IGKV1D-33), complement C4A (Rodgers blood group)(C4A), immunoglobulin kappa variable 3-20(IGKV3-20), complement component 4 binding protein alpha(C4BPA), complement factor H(CFH), complement factor B(CFB), complement C5(C5), complement C6(C6), complement C1q C chain(C1QC), immunoglobulin kappa variable 4-1(IGKV4-1), immunoglobulin heavy constant gamma 4 (G4m marker)(IGHG4)	12.22222	5.62E-10	17.60232	1.15E-07
GO:0002684~positive regulation of immune system process	vascular endothelial growth factor C(VEGFC), immunoglobulin kappa variable 1D-33(IGKV1D-33), interleukin 1 beta(IL1B), complement factor H(CFH), C-X-C motif chemokine ligand 5(CXCL5), C-X-C motif chemokine ligand 1(CXCL1), complement C1q C chain(C1QC), bone marrow stromal cell antigen 1(BST1), C-C motif chemokine ligand 2(CCL2), immunoglobulin kappa variable 4-1(IGKV4-1), immunoglobulin heavy constant gamma 4 (G4m marker)(IGHG4), complement C4A (Rodgers blood group)(C4A), immunoglobulin kappa variable 3-20(IGKV3-20), interleukin 6(IL6), complement component 4 binding protein alpha(C4BPA), peptidoglycan recognition protein 2(PGLYRP2), complement factor B(CFB), intercellular adhesion molecule 1(ICAM1), complement C5(C5), C-X-C motif chemokine ligand 8(CXCL8), complement C6(C6), hemopexin(HPX)	24.44444	1.87E-08	4.290566	2.3E-06
GO:0002443~leukocyte mediated immunity	immunoglobulin kappa variable 1D-33(IGKV1D-33), interleukin 1 beta(IL1B), C-X-C motif chemokine ligand 5(CXCL5), complement C1q C chain(C1QC), immunoglobulin kappa variable 4-1(IGKV4-1), immunoglobulin heavy constant gamma 4 (G4m marker)(IGHG4), complement C4A (Rodgers blood group)(C4A), immunoglobulin kappa variable 3-20(IGKV3-20), interleukin 6(IL6), complement component 4 binding protein	15.55556	4.4E-08	7.36277	4.51E-06

	alpha(C4BPA), complement C5(C5), intercellular adhesion molecule 1(ICAM1), complement C6(C6), hemopexin(HPX)				
GO:0002697~regulation of immune effector process	interleukin 1 beta(IL1B), complement factor H(CFH), C-C motif chemokine ligand 2(CCL2), apolipoprotein A1(APOA1), apolipoprotein A2(APOA2), complement C4A (Rodgers blood group)(C4A), interleukin 6(IL6), complement component 4 binding protein alpha(C4BPA), peptidoglycan recognition protein 2(PGLYRP2), complement factor B(CFB), complement C5(C5), complement C6(C6), hemopexin(HPX)	14.44444	1.35E-07	7.488989	1.19E-05
GO:0050776~regulation of immune response	immunoglobulin kappa variable 1D-33(IGKV1D-33), interleukin 1 beta(IL1B), alpha-1-microglobulin/bikunin precursor(AMBP), complement factor H(CFH), integrin subunit beta 1(ITGB1), complement C1q C chain(C1QC), immunoglobulin kappa variable 4-1(IGKV4-1), immunoglobulin heavy constant gamma 4 (G4m marker)(IGHG4), apolipoprotein A1(APOA1), complement C4A (Rodgers blood group)(C4A), immunoglobulin kappa variable 3-20(IGKV3-20), apolipoprotein A2(APOA2), interleukin 6(IL6), complement component 4 binding protein alpha(C4BPA), peptidoglycan recognition protein 2(PGLYRP2), complement factor B(CFB), intercellular adhesion molecule 1(ICAM1), complement C5(C5), complement C6(C6), hemopexin(HPX)	22.22222	2.17E-07	4.105805	1.67E-05
GO:0002250~adaptive immune response	immunoglobulin kappa variable 1D-33(IGKV1D-33), interleukin 1 beta(IL1B), complement C1q C chain(C1QC), immunoglobulin kappa variable 4-1(IGKV4-1), immunoglobulin heavy constant gamma 4 (G4m marker)(IGHG4), complement C4A (Rodgers blood group)(C4A), immunoglobulin kappa variable 3-20(IGKV3-20), interleukin 6(IL6), complement component 4 binding protein alpha(C4BPA), complement C5(C5), intercellular adhesion molecule 1(ICAM1), joining chain of multimeric IgA and IgM(JCHAIN), complement C6(C6), hemopexin(HPX)	15.55556	2.98E-07	6.240824	2.03E-05
GO:0050778~positive regulation of immune response	immunoglobulin kappa variable 1D-33(IGKV1D-33), interleukin 1 beta(IL1B), complement factor H(CFH), complement C1q C chain(C1QC), immunoglobulin kappa variable 4-1(IGKV4-1), immunoglobulin heavy constant gamma 4 (G4m marker)(IGHG4), complement C4A (Rodgers blood group)(C4A), immunoglobulin kappa variable 3-20(IGKV3-20), interleukin 6(IL6), complement component 4 binding protein alpha(C4BPA), peptidoglycan recognition protein 2(PGLYRP2), complement factor B(CFB), complement C5(C5), complement C6(C6), hemopexin(HPX)	16.66667	1.3E-05	4.105805	0.000445
GO:0002685~regulation of leukocyte migration	vascular endothelial growth factor C(VEGFC), interleukin 6(IL6), C-X-C motif chemokine ligand 5(CXCL5), intercellular adhesion molecule 1(ICAM1), C-X-C motif chemokine ligand 1(CXCL1), complement C5(C5), C-X-C motif chemokine ligand 8(CXCL8), C-C motif chemokine ligand 2(CCL2)	8.888889	1.85E-05	9.601268	0.0006
GO:0002687~positive regulation of leukocyte migration	vascular endothelial growth factor C(VEGFC), interleukin 6(IL6), C-X-C motif chemokine ligand 5(CXCL5), intercellular adhesion molecule 1(ICAM1), C-X-C motif chemokine ligand 1(CXCL1), C-X-C motif chemokine ligand 8(CXCL8), C-C motif chemokine ligand 2(CCL2)	7.777778	2.39E-05	12.02361	0.000669
GO:0002683~negative regulation of immune system process	apolipoprotein A2(APOA2), follistatin like 3(FSTL3), complement component 4 binding protein alpha(C4BPA), alpha-1-microglobulin/bikunin precursor(AMBP), peptidoglycan recognition protein 2(PGLYRP2), amyloid P component, serum(APCS), complement C5(C5), complement C1q C chain(C1QC), C-C motif chemokine ligand 2(CCL2), NME/NM23 nucleoside diphosphate kinase 1(NME1), apolipoprotein A1(APOA1)	12.22222	3.74E-05	5.307917	0.000921
GO:0030595~leukocyte chemotaxis	vascular endothelial growth factor C(VEGFC), interleukin 6(IL6), interleukin 1 beta(IL1B), C-X-C motif chemokine ligand 5(CXCL5), C-X-C motif chemokine ligand 1(CXCL1), complement C5(C5), C-X-C motif chemokine ligand 8(CXCL8), C-C motif chemokine ligand 2(CCL2)	8.888889	7.45E-05	7.720607	0.001697
GO:0045087~innate immune response	complement factor H(CFH), amyloid P component, serum(APCS), complement C1q C chain(C1QC), C-C motif chemokine ligand 2(CCL2), immunoglobulin heavy constant gamma 4 (G4m marker)(IGHG4), complement C4A (Rodgers blood group)(C4A), complement component 4 binding protein alpha(C4BPA), peptidoglycan recognition protein 2(PGLYRP2), complement factor B(CFB), complement C5(C5), intercellular adhesion molecule 1(ICAM1), joining chain of multimeric IgA and IgM(JCHAIN), complement C6(C6), histone cluster 2 H2B family member e(HIST2H2BE), hemopexin(HPX)	16.66667	0.000116	3.359295	0.0023

GO:0097529~myeloid leukocyte migration	vascular endothelial growth factor C(VEGFC), interleukin 6(IL6), interleukin 1 beta(IL1B), C-X-C motif chemokine ligand 1(CXCL1), complement C5(C5), C-X-C motif chemokine ligand 8(CXCL8), C-C motif chemokine ligand 2(CCL2)	7.777778	0.000195	8.242598	0.003532
GO:0050777~negative regulation of immune response	apolipoprotein A2(APOA2), complement component 4 binding protein alpha(C4BPA), peptidoglycan recognition protein 2(PGLYRP2), alpha-1-microglobulin/bikunin precursor(AMBP), apolipoprotein A1(APOA1)	5.555556	0.00409	7.610761	0.041237
GO:0002699~positive regulation of immune effector process	interleukin 6(IL6), interleukin 1 beta(IL1B), complement C6(C6), C-C motif chemokine ligand 2(CCL2), hemopexin(HPX)	5.555556	0.011123	5.708071	0.079545
GO:0002377~immunoglobulin production	interleukin 6(IL6), immunoglobulin kappa variable 4-1(IGKV4-1), polypeptide N-acetyl-galacto-saminyltransferase 2(GALNT2), hemopexin(HPX)	4.444444	0.014289	7.80103	0.094489
GO:0031349~positive regulation of defense response	interleukin 6(IL6), interleukin 1 beta(IL1B), peptidoglycan recognition protein 2(PGLYRP2), kallikrein B1(KLKB1), C-C motif chemokine ligand 2(CCL2), low density lipoprotein receptor(LDLR), hemopexin(HPX)	7.777778	0.019619	3.260132	0.113338

Table S10: Biological process gene ontology (GO:BP) level 3, wounding subset identified through Database for Annotation, Visualization, and Integrated Discovery (DAVID) v6.8 of secreted proteins expressed uniquely in conditional media of human ventricular cardiac fibroblasts treated with IL-1β (10ng/mL). Multiplicity is controlled through Benjamini-Hochberg test.

Term	Gene Name	%	P Value	Fold Enrichment	Benjamini-Hochberg
GO:0009611~response to wounding	tropomyosin 1 (alpha)(TPM1), hemoglobin subunit beta(HBB), pyruvate kinase, muscle(PKM), amyloid P component, serum(APCS), kallikrein B1(KLKB1), C-C motif chemokine ligand 2(CCL2), enolase 3(ENO3), annexin A2(ANXA2), apolipoprotein A1(APOA1), serpin family A member 1(SERPINA1), interleukin 6(IL6), kininogen 1(KNG1)	13.33333	0.000603	3.461782	0.008429
GO:1903035~negative regulation of response to wounding	kallikrein B1(KLKB1), kininogen 1(KNG1), amyloid P component, serum(APCS), annexin A2(ANXA2)	4.44444	0.005569	11.01322	0.049641

Table S11: Biological process gene ontology (GO:BP) level 3, extracellular matrix subset identified through Database for Annotation, Visualization, and Integrated Discovery (DAVID) v6.8 of secreted proteins expressed uniquely in conditional media of human ventricular cardiac fibroblasts treated with IL-1β (10ng/mL). Multiplicity is controlled through Benjamini-Hochberg test.

Term	Gene Name	%	P Value	Fold Enrichment	Benjamini-Hochberg
GO:0043062~extracellular structure organization	heat shock protein family A (Hsp70) member 8(HSPA8), ABI family member 3 binding protein (ABI3BP), versican (VCAN), intercellular adhesion molecule 1(ICAM1), integrin subunit beta 1(ITGB1), collagen type V alpha 2 chain (COL5A2), kallikrein B1(KLKB1), transthyretin(TTR), peroxidasin(PXDN), annexin A2(ANXA2), collagen type VIII alpha 1 chain(COL8A1)	12.22222	1.06E-05	6.147677	0.000384

Table S12: List of metabolites that are differentially expressed in the conditioned media of primary human ventricular cardiac fibroblasts treated with IL-1β (10ng/mL)

Metabolite	Expression	HMDA Class	Role
N-acetyl spermine	Higher	Polyamine	Cell growth and differentiation, androgenic effects of polyamines
Spermidine	Higher	Polyamine	Inhibits pro-inflammatory cytokine synthesis, Induction of autophagy and longevity
N6-Acetyl-L-lysine	Higher	Acetylated amino acid	Biomarker of active genes, stroke risk
Acetyllysine	Lower	Acetylated amino-acid	Marker of exercise incapacity in patients with HF
N-acetyl-glucosamine-1-phosphate	Higher	n-acyl alpha hexosamines	Amino sugar metabolism
Glutathione	Higher	Antioxidant-waste disposal system	Glutathione induced immune stimulatory activity
Adenosine	Lower	Purine nucleoside	Endogenous regulator of innate immunity, coronary vasodilator
Nicotinamide Riboside	Higher	Nicotinate and nicotinamide metabolism	Nicotinamide decrease IL-8 production
D-sedoheptulose-1-7-phosphate	Lower	Hexose phosphates	type III secretion system dependent NFKB activation
p-aminobenzoate	Lower	organic compound	Essential nutrient for bacteria and called vitamin Bx
2-oxo-4-methylthiobutanoate	Higher	Thia fatty acid	IL-3 dependent potent inducer of apoptosis
1,3-diphosphateglycerate	Lower	3-carbon organic molecule	Intermediate of glycolysis during respiration
Citrate-isocitrate	Lower	Tricarboxylic acids and derivatives	Citric acid cycle
Isocitrate	Higher	Tricarboxylic acids and derivatives	Citric acid cycle
Oxaloacetate	Lower	short chain keto acid	oxaloacetate can be converted to citric acid
Succinate	Lower	dicarboxylic acid	Complex II of the electron transport chain
Lactate	Higher	Produced in the muscle	Energy source for the fibers of the heart, Lactate-pyruvate ratio is an indicator of oxidative stress
Citrate	Lower	Energy supply to the body	
2,3-dihydroxybenzoic acid	Lower	Human benzoic acid intermediate	intracellular iron deposition and tissue fibrosis
Cholic acid	Lower	primary bile acid	Facilitates fat absorption and cholesterol excretion
Pyroglutamic acid	Lower	Cyclized derivative of L-glutamic acid	Metabotoxin that causes adverse health effects
Atrolactic acid	Higher		

Table S13: Metabolite concentrations in the conditioned media of IL-1β treated primary human ventricular cardiac fibroblasts (P5). The p values represent two sided Student t test (P value threshold 0.05) and the FDR values calculated from parento scaling normalized data. The mean peak values representing the concentrations of metabolites were calculated from the raw data. FDR is the false discovery rate.

KEGG ID	Metabolite names	Mean peak area intensity +/- SD (Veh)	Mean peak area intensity +/-SD (IL-1β)	Standard deviation (Veh)	Standard deviation (IL-1β)	Fold change (IL-1β/Veh)	P value	(-logP)	FDR (q value)
C00042	Thiamine pyrophosphate		4971.7		2891.1				
C00408	dTMP		4179.9						
C01026	dTDP-nega		4380.8		2680.7				
C00352	dGMP		4108.9		1163.4				
C00818	D-glucosamine-1- phosphate		7432.9						
C00352	dGTP	6598.3		2865.0					
C00364	dUMP-nega		4955.1						
C00160	GTP-nega	2065.4		583.6					
C03150	O-acetyl-L-serine	5754.5		1125.5					
C00378	trans, trans-farnesyl diphosphate	3717.6		583.4					
C00015	UTP-nega	4693.3		1919.1					
C00568	Phenylpropiolic acid	75714.3	2907872.1	11576.5	4908410.4	38.4	0.4	0.4	0.9
C05382	dTMP-nega	6417.4	27713.6	4444.4	35939.7	4.3	0.4	0.4	0.9
C00493	spermine	2891.8	11517.5	585.3	10537.9	4.0	0.4	0.4	0.9
C00167	Uric acid	2489.1	8204.5	7.3	4627.5	3.3	0.2	0.7	0.9
C00705	deoxyadenosine	2065.3	6608.9	583.5	2337.6	3.2	0.1	0.9	0.9
C05463	thymine	6254.2	19984.2	128.8	19807.2	3.2	0.4	0.4	0.9
C02504	2-Isopropylmalic acid	72521.7	227037.0	4814.3	257779.9	3.1	0.4	0.4	0.9
C00131	dehydroascorbic acid	7522.3	22906.3	6535.8	16814.3	3.0	0.2	0.7	0.9
C03492	5-methoxytryptophan	2891.0	8670.5	582.2	4086.7	3.0	0.2	0.7	0.9
C00197	3-S-methylthiopropionate	2477.6	7428.4			3.0			

C00465	Pyrophosphate	37180.3	88984.7	14867.3	109895.1	2.4	0.5	0.3	0.9
HMDB02005	N-acetyl spermidine	1660.8	3643.9		2816.1	2.2			
HMDB0002108	N-acetyl spermine	11549.4	25276.4	2851.9	3304.5	2.2	0.0	2.3	0.5
C01180	2-oxo-4- methylthiobutanoate	12347.3	25439.1	5858.9	6388.9	2.1	0.1	1.2	0.9
C00559	deoxyinosine	4126.9	8258.6	1165.6	5839.3	2.0	0.4	0.4	0.9
C04483	deoxyribose-phosphate	3615.0	7186.8	748.0	2044.7	2.0	0.1	0.8	0.9
HMDB00734	Kynurenic acid	2025905.7	3989798.0	1315608.2	3652608.6	2.0	0.4	0.4	0.9
C00719	biotin	46310.7	89300.0	7237.8	55766.8	1.9	0.3	0.6	0.9
C05852	hypoxanthine	4955.3	9451.3	1655.2	6602.7	1.9	0.3	0.5	0.9
C00158	coenzyme A-posi	2477.5	4719.2		1214.7	1.9			
	orotate	71855.9	135862.4	10838.3	167204.0	1.9	0.5	0.3	0.9
C00049	Atrolactic acid	37916.4	64371.3	13937.3	6295.8	1.7	0.0	1.4	0.9
C00332	Acetyllysine	9619.4	16274.3	3739.2	12015.7	1.7	0.4	0.4	0.9
C00061	fructose-6-phosphate	5367.8	9029.1	2925.5	2388.3	1.7	0.3	0.5	0.9
C00026	allantoin	16184.1	26647.1	8184.3	10594.0	1.6	0.2	0.6	0.9
C00356	3-hydroxybuterate	13454.6	21576.4	7533.7	1480.3	1.6	0.1	0.9	0.9
C00016	folate	15133.8	24166.1	2663.9	11972.4	1.6	0.3	0.6	0.9
C00438	Nicotinamide Riboside	7432.0	11326.9	1649.8	7112.1	1.5	0.4	0.4	0.9
C00882	D-gluconate	3302.7	4973.1		2382.0	1.5			
C00463	isocitrate	8744.2	12709.3	1181.3	2535.4	1.5	0.1	1.2	0.9
C00065	spermidine	99278.5	144242.8	10511.3	19851.1	1.5	0.0	1.6	0.8
C00198	glucose-6-phosphate	39261.6	56600.9	13324.8	17064.0	1.4	0.3	0.5	0.9
C02918	N-acetyl-glucosamine-1-phosphate	15951.1	21545.5	14816.4	11516.4	1.4	0.6	0.2	1.0
C00294	Kynurenine	7570.7	10146.8	2108.0	4575.4	1.3	0.5	0.3	0.9
C00447	sn-glycerol-3-phosphate	111168.2	142903.5	42611.2	41244.6	1.3	0.4	0.4	0.9
C00120	carnitine	128962.3	164318.6	13057.3	96747.9	1.3	0.6	0.2	0.9
C00311	lactate	43867545.6	55529809.0	15572477.1	20366790.9	1.3	0.5	0.3	0.9
C00078	UMP	3280.4	4130.3			1.3			

C03406	malate	335237.8	420964.6	70765.7	238599.8	1.3	0.6	0.2	0.9
C01089	3-methylphenylacetic acid	12651.8	15859.1	7557.7	10263.6	1.3	0.7	0.2	1.0
C03722	S-adenosyl-L-Homocysteine-posi	4955.1	6193.9		1752.2	1.2			
C01236	D-glyceraldehdye-3- phosphate	4438.5	5466.9	1708.4	366.9	1.2	0.4	0.5	0.9
C00295	pantothenate	668847.6	821713.7	132994.8	149481.3	1.2	0.3	0.6	0.9
HMDB01107	acetoacetate	91474.3	111640.6	18913.6	31326.2	1.2	0.4	0.4	0.9
C02494	1-Methyladenosine	6096.6	7418.5	2533.9	2408.3	1.2	0.5	0.3	0.9
C00361	D-glucono-lactone-6- phosphate	8315.9	10098.4	1516.8	4820.6	1.2	0.6	0.2	0.9
C00079	phosphocreatine	319775.2	386385.4	32460.9	76082.4	1.2	0.2	0.6	0.9
C00956	anthranilate	15968.5	19135.1	9801.9	2403.8	1.2	0.6	0.2	1.0
C00860	homocysteine	11380.9	13628.1	582.6	4090.9	1.2	0.4	0.4	0.9
C02571	acetylphosphate	41510.2	49631.3	41607.1		1.2			
C00570	cholesterol	59910.2	71345.5	11868.4	29167.1	1.2	0.6	0.2	0.9
C00213	S-methyl-5-thioadenosine	2117.0	2512.9	560.2		1.2			
C00668	glutathione	19267.5	22805.6	2827.5	14928.9	1.2	0.7	0.2	1.0
HMDB0002092	L-arginino-succinate	30334.9	35286.2	4816.1	6679.2	1.2	0.4	0.4	0.9
C16463	cysteine sulfinate	5229.4	6063.6	515.5	5611.1	1.2	0.8	0.1	1.0
HMDB00746	hydroxyproline	21438.5	24848.8	11121.6	16170.3	1.2	0.8	0.1	1.0
C00118	dihydroorotate	115973.9	133286.9	46887.5	76175.6	1.1	0.8	0.1	1.0
C00086	xanthosine	10449.5	11556.1	5489.5	7048.4	1.1	0.8	0.1	1.0
C00170	S-ribosyl-L-homocysteine-posi	649413.2	712348.7	59301.2	98611.2	1.1	0.4	0.4	0.9
C00882	dGDP-nega	4541.6	4955.2	1752.1		1.1			
C01144	3-phosphoglycerate	11909.9	12993.6	3109.3	4219.7	1.1	0.7	0.1	1.0
C00130	inosine	286305.8	309446.0	32473.8	71274.3	1.1	0.6	0.2	1.0
C00864	phenylpyruvate	12626.4	13632.0	5203.5	9881.9	1.1	0.9	0.1	1.0

HMDB00991	2-Aminooctanoic acid	8918.7	9549.6	4146.6	2349.3	1.1	0.8	0.1	1.0
C15608	fructose-1,6-bisphosphate	24438.8	26137.2	7521.5	8021.3	1.1	0.8	0.1	1.0
C02727	N-acetyl-glutamine	64757.5	69011.1	73113.2	91643.7	1.1	1.0	0.0	1.0
C00072	aspartate	2830883.4	2988371.4	698971.8	383149.0	1.1	0.7	0.1	1.0
C00334	4-Pyridoxic acid	24182.9	25527.6	16473.9	7577.5	1.1	0.9	0.0	1.0
C00063	cysteine	112944.5	118985.8	33294.7	29892.2	1.1	0.8	0.1	1.0
C00024	aconitate	112225.3	117654.5	21906.1	32475.1	1.0	0.8	0.1	1.0
C00299	Xanthurenic acid	298163.1	312326.3	239786.8	254273.1	1.0	0.9	0.0	1.0
C00109	2-oxobutanoate	127614.1	133159.7	30704.5	56372.8	1.0	0.9	0.1	1.0
C00144	guanine	10588.4	11001.8	2682.2	3654.7	1.0	0.9	0.1	1.0
C00064	glutathione disulfide-posi	12032.2	12437.5	3846.4	3441.3	1.0	0.9	0.0	1.0
C00135	homocysteic acid	26430.5	27261.9	15159.1	15282.9	1.0	0.9	0.0	1.0
HMDB06029	N-Acetylputrescine	15708.9	16189.6	2679.5	3520.7	1.0	0.9	0.1	1.0
C02835	Indoleacrylic acid	46067.9	47436.6	25937.6	17503.2	1.0	0.9	0.0	1.0
C00458	deoxyguanosine	6883.0	7071.9	3726.6	2977.6	1.0	0.9	0.0	1.0
C00105	valine	2762540.9	2834181.4	522316.3	567688.0	1.0	0.9	0.1	1.0
C00750	taurine	29801.9	30541.5	4257.1	2544.0	1.0	0.8	0.1	1.0
C01157	Imidazoleacetic acid	489139.2	499923.1	81656.9	59550.0	1.0	0.9	0.1	1.0
C00123	Maleic acid	345604.0	353087.6	95495.8	28571.9	1.0	0.9	0.0	1.0
C00029	Urea	153622.9	156769.9	18448.3	70600.8	1.0	0.9	0.0	1.0
C04677	arginine	52828700.5	53877163.0	4037576.4	8569760.7	1.0	0.9	0.1	1.0
C00575	Cystine	5377824.3	5478109.0	518556.6	2035423.5	1.0	0.9	0.0	1.0
C00360	dCTP-nega	3302.8	3302.7			1.0			
C00024	adenine	404794.4	404666.2	16632.2	81734.9	1.0	1.0	0.0	>0.999999
C01384	Methylcysteine	404794.4	404666.2	16632.2	81734.9	1.0	1.0	0.0	>0.999999
C00345	7-methylguanosine	13767.4	13757.2	3440.5	8020.5	1.0	1.0	0.0	>0.999999
C03539	Taurodeoxycholic acid	107092.1	105712.4	13376.5	32334.9	1.0	0.9	0.0	1.0

C00455	OBP	19455.0	18993.1	2642.8	3295.3	1.0	0.9	0.1	1.0
C00025	glutathione disulfide-nega	36723.2	35776.8	7699.9	8734.9	1.0	0.9	0.0	1.0
C00438	Nicotinamide ribotide	5505.3	5354.8	954.9	2938.9	1.0	0.9	0.0	1.0
PubChem Identifier (KEGG/HMDB)	myo-inositol	15207718.8	14697583.0	2057383.8	1898898.8	1.0	0.8	0.1	1.0
HMDB00563	Phosphorylcholine	64146.8	61875.2	14883.0	29437.9	1.0	0.9	0.0	1.0
C00263	hydroxyphenylpyruvate	830811.3	799691.8	17272.9	52142.2	1.0	0.4	0.4	0.9
C00127	glycerate	828746.1	796036.7	35304.8	172131.9	1.0	0.8	0.1	1.0
C00002	betaine	1070121.4	1023654.5	218533.4	252452.4	1.0	0.8	0.1	1.0
C00117	sarcosine	3900314.9	3726779.4	438769.7	1295396.2	1.0	0.8	0.1	1.0
C01159	2,3-Diphosphoglyceric acid	4308.2	4103.0	912.3		1.0			
C00341	glucose-1-phosphate	33921.8	32074.7	6060.0	7642.9	0.9	0.8	0.1	1.0
C00149	Methionine sulfoxide	87560.1	82631.8	24939.1	17265.0	0.9	0.8	0.1	1.0
C00166	p-hydroxybenzoate	97729.4	91880.7	37840.6	70355.6	0.9	0.9	0.0	1.0
C00082	uracil	76914.5	71680.1	5059.4	1892.1	0.9	0.2	0.8	0.9
HMDB00475	betaine aldehyde	48389.7	44940.7	17369.5	8040.1	0.9	0.8	0.1	1.0
C00242	hexose-phosphate	60960.3	56127.7	5297.6	10027.7	0.9	0.5	0.3	0.9
C00051	glutathione-nega	26298.8	24125.0	4309.5	13131.1	0.9	0.8	0.1	1.0
C00498	alanine	5228301.0	4796127.5	383683.6	1577145.5	0.9	0.7	0.2	1.0
C00021	serine	20054935.7	18395588.8	1144971.0	6324430.2	0.9	0.7	0.2	1.0
C16511	Hydroxyisocaproic acid	510464.3	464367.5	55652.1	68877.3	0.9	0.4	0.4	0.9
C00036	Phenyllactic acid	160473.1	145617.7	26090.4	33411.2	0.9	0.6	0.2	0.9
C00417	Adenylosuccinate	115070.1	104333.9	42837.6	25925.3	0.9	0.7	0.1	1.0
C00035	glucosamine	1235908.6	1116755.1	67073.3	174336.1	0.9	0.3	0.5	0.9
C00670	glyoxylate	15015.9	13550.3	5797.3	4979.6	0.9	0.8	0.1	1.0
C00629	2-dehydro-D-gluconate	21688.6	19534.4	3719.2	6107.0	0.9	0.6	0.2	1.0

C00005	nicotinamide	104498579.0	93850575.0	10852864.8	20082712.0	0.9	0.5	0.3	0.9
C02630	2-hydroxygluterate	503624.0	451181.1	55595.1	41761.9	0.9	0.3	0.6	0.9
C00013	ribose-phosphate	11658.7	10416.3	6560.1	6475.3	0.9	0.8	0.1	1.0
C00103	glutamine	82092538.1	73140380.2	2865570.5	9106056.4	0.9	0.2	0.7	0.9
C00460	Flavone	20425.8	18121.8	6513.3	4029.5	0.9	0.6	0.2	1.0
C00055	Creatinine	21146.8	18716.5	7090.5	7577.5	0.9	0.7	0.1	1.0
C00047	methionine	1226252.7	1085196.8	151808.0	27338.7	0.9	0.2	0.7	0.9
C00279	D-gluconate	131302.5	115926.5	14537.3	3919.9	0.9	0.2	0.8	0.9
C00329	glutamate	9690338.2	8553138.1	1727038.7	1740264.3	0.9	0.5	0.3	0.9
C00362	dihydroxy-acetone- phosphate	8701.8	7667.9	7055.1		0.9			
C00100	Pyroglutamic acid	958870.2	840319.6	37528.3	63907.1	0.9	0.1	1.3	0.9
C00491	cytosine	337420.5	295633.4	45305.2	82136.2	0.9	0.5	0.3	0.9
C00074	proline	31160052.1	26950570.5	4423268.0	4434999.7	0.9	0.3	0.5	0.9
C00387	histidine	63147455.2	54366853.6	7322275.8	11274981.2	0.9	0.3	0.5	0.9
C01152	1-Methyl-Histidine	70150.3	60372.8	19207.4	38344.0	0.9	0.7	0.1	1.0
C00127	Glycerophosphocholine	5686411.0	4885411.6	1696986.5	1828239.0	0.9	0.6	0.2	1.0
C02170	N-acetyl-glucosamine	5782.7	4965.1			0.9			
C00141	2-keto-isovalerate	366764.8	313697.6	88616.7	23274.7	0.9	0.4	0.4	0.9
C01717	leucine-isoleucine	96782912.2	82724090.4	2396428.4	16193482.9	0.9	0.2	0.7	0.9
C00155	Hydroxyphenylacetic acid	92066.5	78585.8	20634.8	63072.3	0.9	0.7	0.1	1.0
C02612	2-Hydroxy-2-methylbutanedioic acid	186310.0	158742.2	20949.0	78646.1	0.9	0.6	0.2	0.9
C00005	Ng,NG-dimethyl-L- arginine	282483.8	239422.7	91068.2	159966.0	0.8	0.7	0.2	1.0
C00224	a-ketoglutarate	5791637.4	4903165.0	452318.0	1020095.9	0.8	0.2	0.6	0.9
C00214	tyrosine	1290795.3	1091576.8	182630.2	159115.4	0.8	0.2	0.6	0.9
C00091	threonine	25234183.0	21309601.7	1044230.8	5610259.0	0.8	0.3	0.5	0.9

C00777	S-adenosyl-L- methioninamine	8330.1	7027.9		2909.9	0.8			
HMDB01864	2-ketohaxanoic acid	14211.5	11963.9	6102.7	5145.8	0.8	0.7	0.2	1.0
C00062	asparagine	7246627.4	6086825.6	467921.7	1141953.8	0.8	0.2	0.7	0.9
C00108	Ascorbic acid	2043146.7	1714385.7	503577.1	886389.7	0.8	0.6	0.2	1.0
C00020	argininosuccinic acid	44621.7	37277.1	9257.8	4595.3	0.8	0.3	0.5	0.9
C00257	D-glucosamine-6- phosphate	4955.3	4130.0			0.8			
C00111	DL-Pipecolic acid	8068006.5	6611906.7	895098.1	1866394.7	0.8	0.3	0.5	0.9
C03626	nicotinate	304839.8	249526.4	76101.3	111833.5	0.8	0.5	0.3	0.9
C00327	creatine	419903.1	339636.4	157894.8	222803.1	0.8	0.6	0.2	1.0
HMDB02222	3-phospho-serine	2068.5	1655.3	584.2	0.1	0.8	0.4	0.4	0.9
C00112	cholesteryl sulfate	25738.0	20506.3	1672.3	19475.2	0.8	0.7	0.2	1.0
C00048	Guanidoacetic acid	390047.7	304627.8	35528.3	87129.6	0.8	0.2	0.7	0.9
C00156	purine	167279.1	130449.1	13371.5	44920.4	0.8	0.2	0.6	0.9
C00041	Aminoadipic acid	529205.7	412010.3	86977.4	191935.6	0.8	0.4	0.4	0.9
C08276	4-phosphopantothenate	3768.7	2932.9	1775.2	1777.1	0.8	0.7	0.2	1.0
C00979	orotidine-5-phosphate	12460.7	9642.7	9018.9	4305.5	0.8	0.7	0.1	1.0
C05512	dephospho-CoA-nega	8670.6	6606.7	7591.2	2335.4	0.8	0.7	0.1	1.0
C00044	guanosine	5379.9	4096.6	566.3	876.9	0.8	0.2	0.8	0.9
C00186	lysine	4025561.1	3046125.2	495674.1	885946.0	0.8	0.2	0.8	0.9
C00504	fumarate	419519.0	317257.2	96207.7	26821.7	0.8	0.2	0.8	0.9
C00624	N-acetyl-L-ornithine	16523.5	12410.6	9718.9	3636.7	0.8	0.5	0.3	0.9
C00188	tryptophan	9100800.7	6668861.2	1232518.8	2902576.3	0.7	0.3	0.6	0.9
C01137	shikimate	10059390.7	7363365.9	6498214.5	7434861.0	0.7	0.7	0.2	1.0
C06369	2-deoxyglucose-6-phosphate	84255.2	61637.5	16652.7	20209.9	0.7	0.2	0.7	0.9
C00100	pyridoxine	1141287.4	834836.3	133669.3	233605.1	0.7	0.1	0.9	0.9
C01879	riboflavin	65769.9	47704.5	13442.2	9184.9	0.7	0.1	0.9	0.9
C02226	citrulline	1421175.4	1024245.4	184904.1	885199.0	0.7	0.5	0.3	0.9
C00253	ornithine	150337.9	108204.9	14488.1	65952.1	0.7	0.3	0.5	0.9

C00106	xanthine	69089.5	49199.1	28962.9	31747.4	0.7	0.5	0.3	0.9
C00356	homoserine	17014.4	12111.4	6323.8	3504.2	0.7	0.3	0.5	0.9
C00083	methylnicotinamide	6287.9	4467.0	2038.5	280.6	0.7	0.3	0.5	0.9
C00134	pyruvate	96006.4	67785.0	32212.7	26437.7	0.7	0.3	0.5	0.9
C00043	uridine	12888.4	9087.1	8236.7	1273.8	0.7	0.5	0.3	0.9
C00153	O8P-O1P	8808.9	6195.2	5977.9	2537.0	0.7	0.5	0.3	0.9
C00526	D-erythrose-4-phosphate	33859.6	23249.9	15597.9	8949.4	0.7	0.4	0.4	0.9
C00104	indole	28360.4	19391.9	7842.7	10130.9	0.7	0.3	0.5	0.9
C01103	phenylalanine	13507914.9	9092541.0	1674663.1	4012913.3	0.7	0.2	0.8	0.9
C00137	N-acetyl-glutamate	81018.0	53410.8	44732.2	85077.3	0.7	0.6	0.2	1.0
C00254	putrescine	21416.2	13740.4	7802.8	9796.6	0.6	0.4	0.4	0.9
C00187	choline	373867286.4	238201833.8	17644489.7	206309267.9	0.6	0.3	0.5	0.9
C01589	Indole-3-carboxylic acid	338185.9	212961.0	114146.8	82865.3	0.6	0.2	0.7	0.9
C00440	6-phospho-D-gluconate	6603.2	4130.4	1176.8	3504.1	0.6	0.4	0.4	0.9
C00337	dimethylglycine	60451143.1	37527151.1	1421161.9	32790243.7	0.6	0.3	0.5	0.9
C00164	Acetylcarnitine DL	42678.9	26105.0	8913.3	14832.0	0.6	0.2	0.8	0.9
C01479	phosphoenolpyruvate	6706.2	4074.0			0.6			
C00534	quinolinate	79790.6	48043.3	57876.0	60230.7	0.6	0.5	0.3	0.9
C00576	Carbamoyl phosphate	151654.7	90784.6	102908.5	57305.9	0.6	0.4	0.4	0.9
C00021	SBP	70103.8	41329.0	13652.9	18017.2	0.6	0.1	1.0	0.9
C00083	Methylmalonic acid	320943.6	185908.2	100520.0	66474.3	0.6	0.1	0.9	0.9
HMDB03320	itaconic acid	357129.6	206283.5	95949.1	136817.2	0.6	0.2	0.7	0.9
C00185	Cholic acid	12321.9	7106.8	1497.0	3846.6	0.6	0.1	1.0	0.9
C00300	cyclic-AMP	82874.5	47625.7	32434.8	16399.8	0.6	0.2	0.8	0.9
C00122	glucono-?-lactone	7176.7	4074.2	847.0		0.6			
C00196	2,3-dihydroxybenzoic acid	91493.3	51473.9	11087.4	27302.1	0.6	0.1	1.1	0.9
C00093	succinate	299961.8	164899.1	25435.1	12191.3	0.5	0.0	2.9	0.2
C00148	Pyridoxamine	4543.6	2425.5	1749.0		0.5			
C00236	1,3-diphopshateglycerate	10876.4	5773.0	2439.4	1863.0	0.5	0.0	1.3	0.9

C00695	citrate	7384821.6	3807418.2	957557.5	2165632.5	0.5	0.1	1.2	0.9
C00140	N-acetyl-L-aspartic acid	4961.5	2524.0			0.5			
C12270	N-Acetyl-L-alanine	146626.2	73479.4	52193.6	47684.6	0.5	0.1	0.8	0.9
C00008	allantoate	7383.3	3650.4		542.1	0.5			
C00258	glycolate	75951.1	37336.0	47589.1	28155.8	0.5	0.3	0.5	0.9
C00073	N6-Acetyl-L-lysine	10563.8	5027.3	1376.4	1269.7	0.5	0.0	1.7	0.8
HMDB00653	Citraconic acid/itaconic acid	326088.9	153454.6	44439.1	180435.3	0.5	0.2	0.7	0.9
C00318	CDP-nega	8451.8	3770.1	4733.6	822.7	0.4	0.3	0.6	0.9
C00022	S-adenosyl-L-homocysteine-nega	14866.3	6606.5	10150.3	1168.4	0.4	0.4	0.4	0.9
C00791	cystathionine	15138.0	6607.9	5613.8		0.4			
C00077	p-aminobenzoate	23980.8	9477.5	12853.3	1842.8	0.4	0.1	0.9	0.9
C02727	adenosine	9019.4	3256.9	4819.8	751.9	0.4	0.1	1.0	0.9
	oxaloacetate	213838.6	72676.7	61963.8	10111.8	0.3	0.0	1.8	0.8
C00114	citrate-isocitrate	16692631.3	5644964.4	920501.0	4359663.8	0.3	0.0	1.9	0.8
HMDB00582	D-sedoheptulose-1-7- phosphate	100424.4	31726.2	40233.3	12853.3	0.3	0.0	1.3	0.9
C02291	cytidine	42962.9	10842.4	21436.0	1281.7	0.3	0.1	1.2	0.9
