# Supplementary material for: IL-1β-mediated adaptive reprogramming of endogenous human cardiac fibroblasts to cells with immune features during fibrotic remodeling
Source: Commun Biol. 2023 Nov 25;6:1200. doi: 10.1038/s42003-023-05463-0 (PMC10673909; doi:10.1038/s42003-023-05463-0)
Supplement: Supplementary file 3 — Description of Additional Supplementary Files [file 42003_2023_5463_MOESM3_ESM.docx]

**Description of Additional Supplementary Files**

**File name:** Supplementary Data 1

**Description:** The source data behind the figures
