# Supplementary material for: IL-1β-mediated adaptive reprogramming of endogenous human cardiac fibroblasts to cells with immune features during fibrotic remodeling
Source: Commun Biol. 2023 Nov 25;6:1200. doi: 10.1038/s42003-023-05463-0 (PMC10673909; doi:10.1038/s42003-023-05463-0)
Supplement: Supplementary file 8 — Reporting Summary [file 42003_2023_5463_MOESM8_ESM.pdf]

nature portfolio

Corresponding author(s): Prof. Henning Boecker

Last updated by author(s): 2023 09 08

Reporting Summary

Nature Portfolio wishes to improve the reproducibility of the work that we publish. This form provides structure for consistency and transparency in reporting. For further information on Nature Portfolio policies, see our Editorial Policies and the Editorial Policy Checklist.

Statistics

For all statistical analyses, confirm that the following items are present in the figure legend, table legend, main text, or Methods section.

n/a	Confirmed	
☐	☒	The exact sample size (n) for each experimental group/condition, given as a discrete number and unit of measurement
☐	☒	A statement on whether measurements were taken from distinct samples or whether the same sample was measured repeatedly
☐	☒	The statistical test(s) used AND whether they are one- or two-sided<br>*Only common tests should be described solely by name; describe more complex techniques in the Methods section.*
☐	☒	A description of all covariates tested
☐	☒	A description of any assumptions or corrections, such as tests of normality and adjustment for multiple comparisons
☐	☒	A full description of the statistical parameters including central tendency (e.g. means) or other basic estimates (e.g. regression coefficient) AND variation (e.g. standard deviation) or associated estimates of uncertainty (e.g. confidence intervals)
☐	☒	For null hypothesis testing, the test statistic (e.g. F, t, r) with confidence intervals, effect sizes, degrees of freedom and P value noted<br>*Give P values as exact values whenever suitable.*
☒	☐	For Bayesian analysis, information on the choice of priors and Markov chain Monte Carlo settings
☐	☒	For hierarchical and complex designs, identification of the appropriate level for tests and full reporting of outcomes
☐	☒	Estimates of effect sizes (e.g. Cohen's d, Pearson's r), indicating how they were calculated

Our web collection on statistics for biologists contains articles on many of the points above.

Software and code

Policy information about availability of computer code

Data collection	The computerized spatial object memory task was adapted from previous studies (e.g. Kunz et al, Science 2015: https://doi.org:10.1126/science.aac8128). The paradigm was developed using Unreal Engine 2 (Epic Games, Cary, NC, USA) with customized code. Source codes can be made available on request.
Data analysis	Imaging data were processed using FreeSurfer 6.0 (for longitudinal registration/ segmentation) and 7.0 (for subsequent hippocampal subfield segmentation).<br>Demographic, behavioral, physiological and volumetric data were analyzed with SPSS 27.0 (IBM Corp, Armonk, NY).<br>Source codes for command scripts used will be made available on request.

For manuscripts utilizing custom algorithms or software that are central to the research but not yet described in published literature, software must be made available to editors and reviewers. We strongly encourage code deposition in a community repository (e.g. GitHub). See the Nature Portfolio guidelines for submitting code & software for further information.

Data

Policy information about availability of data

All manuscripts must include a data availability statement. This statement should provide the following information, where applicable:

- Accession codes, unique identifiers, or web links for publicly available datasets
- A description of any restrictions on data availability
- For clinical datasets or third party data, please ensure that the statement adheres to our policy

Data can only be made available after contacting participating volunteers and obtaining their consent to submit data in pseudonymized form (as per ethics' approval). Depending on the decision of the volunteers, this may result in smaller samples.

Human research participants

Policy information about studies involving human research participants and Sex and Gender in Research.

Reporting on sex and gender	Information regarding sex of the participating individuals (males and females, based on self-report) were collected, and used as a covariate in statistical analysis. As we had no a priori hypotheses regarding sex-specific effects, sex was not used as a stratification variable in the randomization process. Due to the limited sample sizes, we did also not perform sex-stratified group analyses.
Population characteristics	The study examined healthy young adults (age 18-35 years) without prior history as competitive athletes and/or regular physical exercise training in the previous 2 years. Exclusion criteria were psychiatric, neurologic, or cardiovascular diseases (current or in the past). Before each MRI session, participants were informed and screened for contraindications. Female participants were additionally tested for pregnancy before each MR examination.<br>Detailed characteristics are given in Table 1.
Recruitment	Volunteers were recruited through social media and via flyers at the local university.
Ethics oversight	The study was approved by the Ethics Committee at the Medical Faculty of the Rheinische Friedrich-Wilhelms-Universität Bonn (no. 370/15) and conducted according to national legislation and the Declaration of Helsinki.

Note that full information on the approval of the study protocol must also be provided in the manuscript.

Field-specific reporting

Please select the one below that is the best fit for your research. If you are not sure, read the appropriate sections before making your selection.

☒ Life sciences ☐ Behavioural & social sciences ☐ Ecological, evolutionary & environmental sciences

For a reference copy of the document with all sections, see nature.com/documents/nr-reporting-summary-flat.pdf

Life sciences study design

All studies must disclose on these points even when the disclosure is negative.

Sample size	This study had a final sample size of N = 27 subjects with N = 17 in the exercise intervention group and N = 10 in the control group. These subjects were scanned four times each at two-month intervals for a total 6-month time-period.
Data exclusions	Detailed description of data exclusions is provided in earlier studies on the basis of the same cohort (e.g. Upadhyay al. (2022), Front Aging Neurosci 14, 951022, https://doi.org:10.3389/fnagi.2022.951022).<br><br>Briefly, N=57 participants were assessed for eligibility after giving their informed consent. Of these, N=9 dropped out during these assessments due to loss of interest. The remaining N=48 subjects were randomized into the intervention group (IG, N=32) and control group (CG, N=16).<br>During the course of the study, further participants dropped out for various reasons, including panic attacks or new MR contraindications which precluded MR examinations, loss of interest, or injuries caused by private activities, leaving N=21 IG and N=11 CG participants who completed the intervention phase.<br>For the present analyses, N=4 IG and N=1 CG participant had excluded retrospectively, due to the development of depressive symptoms during the intervention, missing 3T MR data, insufficient adherence to the intervention schedule, and lack of compliance in the Spatial Object Memory Task.
Replication	Due to the limited sample size, all available datasets were used, and no replication was performed in separate cohorts. However, further studies with larger sample sizes are warrented.
Randomization	Subjects were randomly assigned to either the exercise intervention group or the control group. Due to the expected higher dropout rate in the exercise intervention group subjects were assigned in a 2:1 ratio (intervention : control) using the sequential list randomization method.

Blinding	As by nature, subjects could not be blinded with regard to being randomized to the exercise intervention group or the control group.

Reporting for specific materials, systems and methods

We require information from authors about some types of materials, experimental systems and methods used in many studies. Here, indicate whether each material, system or method listed is relevant to your study. If you are not sure if a list item applies to your research, read the appropriate section before selecting a response.

Materials & experimental systems

n/a	Involved in the study
☐	☐ Antibodies
☐	☐ Eukaryotic cell lines
☐	☐ Palaeontology and archaeology
☐	☐ Animals and other organisms
☐	☐ Clinical data
☐	☐ Dual use research of concern

Methods

n/a	Involved in the study
☐	☐ ChIP-seq
☐	☐ Flow cytometry
☐	☒ MRI-based neuroimaging

Antibodies

Antibodies used	*Describe all antibodies used in the study; as applicable, provide supplier name, catalog number, clone name, and lot number.*
Validation	*Describe the validation of each primary antibody for the species and application, noting any validation statements on the manufacturer's website, relevant citations, antibody profiles in online databases, or data provided in the manuscript.*

Eukaryotic cell lines

Policy information about cell lines and Sex and Gender in Research

Cell line source(s)	*State the source of each cell line used and the sex of all primary cell lines and cells derived from human participants or vertebrate models.*
Authentication	*Describe the authentication procedures for each cell line used OR declare that none of the cell lines used were authenticated.*
Mycoplasma contamination	*Confirm that all cell lines tested negative for mycoplasma contamination OR describe the results of the testing for mycoplasma contamination OR declare that the cell lines were not tested for mycoplasma contamination.*
Commonly misidentified lines (See ICLAC register)	*Name any commonly misidentified cell lines used in the study and provide a rationale for their use.*

Palaeontology and Archaeology

Specimen provenance	*Provide provenance information for specimens and describe permits that were obtained for the work (including the name of the issuing authority, the date of issue, and any identifying information). Permits should encompass collection and, where applicable, export.*
Specimen deposition	*Indicate where the specimens have been deposited to permit free access by other researchers.*
Dating methods	*If new dates are provided, describe how they were obtained (e.g. collection, storage, sample pretreatment and measurement), where they were obtained (i.e. lab name), the calibration program and the protocol for quality assurance OR state that no new dates are provided.*

☐ Tick this box to confirm that the raw and calibrated dates are available in the paper or in Supplementary Information.

Ethics oversight	*Identify the organization(s) that approved or provided guidance on the study protocol, OR state that no ethical approval or guidance was required and explain why not.*

Note that full information on the approval of the study protocol must also be provided in the manuscript.

Animals and other research organisms

Policy information about studies involving animals; ARRIVE guidelines recommended for reporting animal research, and Sex and Gender in Research

Laboratory animals	*For laboratory animals, report species, strain and age OR state that the study did not involve laboratory animals.*
Wild animals	*Provide details on animals observed in or captured in the field; report species and age where possible. Describe how animals were caught and transported and what happened to captive animals after the study (if killed, explain why and describe method; if released, say where and when) OR state that the study did not involve wild animals.*
Reporting on sex	*Indicate if findings apply to only one sex; describe whether sex was considered in study design, methods used for assigning sex. Provide data disaggregated for sex where this information has been collected in the source data as appropriate; provide overall numbers in this Reporting Summary. Please state if this information has not been collected. Report sex-based analyses where performed, justify reasons for lack of sex-based analysis.*
Field-collected samples	*For laboratory work with field-collected samples, describe all relevant parameters such as housing, maintenance, temperature, photoperiod and end-of-experiment protocol OR state that the study did not involve samples collected from the field.*
Ethics oversight	*Identify the organization(s) that approved or provided guidance on the study protocol, OR state that no ethical approval or guidance was required and explain why not.*

Note that full information on the approval of the study protocol must also be provided in the manuscript.

Clinical data

Policy information about clinical studies

All manuscripts should comply with the ICMJE guidelines for publication of clinical research and a completed CONSORT checklist must be included with all submissions.

Clinical trial registration	*Provide the trial registration number from ClinicalTrials.gov or an equivalent agency.*
Study protocol	*Note where the full trial protocol can be accessed OR if not available, explain why.*
Data collection	*Describe the settings and locales of data collection, noting the time periods of recruitment and data collection.*
Outcomes	*Describe how you pre-defined primary and secondary outcome measures and how you assessed these measures.*

Dual use research of concern

Policy information about dual use research of concern

Hazards

Could the accidental, deliberate or reckless misuse of agents or technologies generated in the work, or the application of information presented in the manuscript, pose a threat to:

No	Yes	
☒	☐	Public health
☒	☐	National security
☒	☐	Crops and/or livestock
☒	☐	Ecosystems
☒	☐	Any other significant area

Experiments of concern

Does the work involve any of these experiments of concern:

No	Yes	
☒	☐	Demonstrate how to render a vaccine ineffective
☒	☐	Confer resistance to therapeutically useful antibiotics or antiviral agents
☒	☐	Enhance the virulence of a pathogen or render a nonpathogen virulent
☒	☐	Increase transmissibility of a pathogen
☒	☐	Alter the host range of a pathogen
☒	☐	Enable evasion of diagnostic/detection modalities
☒	☐	Enable the weaponization of a biological agent or toxin
☒	☐	Any other potentially harmful combination of experiments and agents

ChIP-seq

Data deposition

☐ Confirm that both raw and final processed data have been deposited in a public database such as GEO.

☐ Confirm that you have deposited or provided access to graph files (e.g. BED files) for the called peaks.

Data access links<br>*May remain private before publication.*	*For "Initial submission" or "Revised version" documents, provide reviewer access links. For your "Final submission" document, provide a link to the deposited data.*
Files in database submission	*Provide a list of all files available in the database submission.*
Genome browser session<br>(e.g. UCSC)	*Provide a link to an anonymized genome browser session for "Initial submission" and "Revised version" documents only, to enable peer review. Write "no longer applicable" for "Final submission" documents.*

Methodology

Replicates	*Describe the experimental replicates, specifying number, type and replicate agreement.*
Sequencing depth	*Describe the sequencing depth for each experiment, providing the total number of reads, uniquely mapped reads, length of reads and whether they were paired- or single-end.*
Antibodies	*Describe the antibodies used for the ChIP-seq experiments; as applicable, provide supplier name, catalog number, clone name, and lot number.*
Peak calling parameters	*Specify the command line program and parameters used for read mapping and peak calling, including the ChIP, control and index files used.*
Data quality	*Describe the methods used to ensure data quality in full detail, including how many peaks are at FDR 5% and above 5-fold enrichment.*
Software	*Describe the software used to collect and analyze the ChIP-seq data. For custom code that has been deposited into a community repository, provide accession details.*

Flow Cytometry

Plots

Confirm that:

☐ The axis labels state the marker and fluorochrome used (e.g. CD4-FITC).

☐ The axis scales are clearly visible. Include numbers along axes only for bottom left plot of group (a 'group' is an analysis of identical markers).

☐ All plots are contour plots with outliers or pseudocolor plots.

☐ A numerical value for number of cells or percentage (with statistics) is provided.

Methodology

Sample preparation	*Describe the sample preparation, detailing the biological source of the cells and any tissue processing steps used.*
Instrument	*Identify the instrument used for data collection, specifying make and model number.*

Software	*Describe the software used to collect and analyze the flow cytometry data. For custom code that has been deposited into a community repository, provide accession details.*
Cell population abundance	*Describe the abundance of the relevant cell populations within post-sort fractions, providing details on the purity of the samples and how it was determined.*
Gating strategy	*Describe the gating strategy used for all relevant experiments, specifying the preliminary FSC/SSC gates of the starting cell population, indicating where boundaries between "positive" and "negative" staining cell populations are defined.*

☐ Tick this box to confirm that a figure exemplifying the gating strategy is provided in the Supplementary Information.

Magnetic resonance imaging

Experimental design

Design type	Structural MRI
Design specifications	Per subject one structural MRI was acquired four times each at two-month intervals, for a total 6-month time-period.
Behavioral performance measures	Participants performed a computerized spatial object memory task outside of the scanner (i.e. no task fMRI) where the position of objects had to be learned and recollected while navigating in a 3D virtual arena. Spatial memory performance was operationalized by the mean drop error, i.e. the Euclidian distance between the response location and the correct object location, averaged over 160 trials per session.

Acquisition

Imaging type(s)	Structural MRI:
Field strength	3T
Sequence & imaging parameters	MRI scans were performed at a 3T Siemens Magnetom Skyra System with a 32-channel head-coil. Anatomical T1 weighted (T1w) images were acquired using an in-house developed MP-RAGE sequence with 1x3z1 CAIPIRINHA and elliptical sampling with the following specifications: sagittal slice orientation, voxel size = 1 x 1 x 1 mm, field-of-view = 192 x 192 x 144 mm, TR = 2.5 s, TI = 1.1 s, TE = 5 ms, flip angle = 7°, total scan duration: 2 minutes 53 seconds.
Area of acquisition	whole brain

Diffusion MRI ☐ Used ☒ Not used

Preprocessing

Preprocessing software	FreeSurfer 6.0 and 7.0 (http://surfer.nmr.mgh.harvard.edu/)
Normalization	Normalization to MNI space for brain parcellation purposes
Normalization template	Desikan-Killiany
Noise and artifact removal	N4 bias correction
Volume censoring	n/a

Statistical modeling & inference

Model type and settings	linear mixed effects (LME) model
Effect(s) tested	In case of significant effects (time, group, or time by group interaction), post hoc tests between time points within each group were conducted. Between-group comparisons were performed for each time point separately but also for the change in volume over time (Delta T2 minus T0 (DT2T0), DT4T0, and DT6T0).

Specify type of analysis: ☐ Whole brain ☒ ROI-based ☐ Both

Anatomical location(s)	hippocampus
Statistic type for inference (See Eklund et al. 2016)	n/a
Correction	P-values were adjusted using Bonferroni correction. Results of post hoc tests were considered significant when $p < 0.05$ (two-sided) and are reported with Cohen´s d as an effect size

Models & analysis

n/a	Involved in the study
☒	☐ Functional and/or effective connectivity
☒	☐ Graph analysis
☒	☐ Multivariate modeling or predictive analysis
